# A multiband NIR upconversion core-shell design for enhanced light harvesting of silicon solar cells

**DOI:** 10.1038/s41377-024-01661-5

**Published:** 2024-11-25

**Authors:** Yue Wang, Wen Xu, Haichun Liu, Yuhan Jing, Donglei Zhou, Yanan Ji, Jerker Widengren, Xue Bai, Hongwei Song

**Affiliations:** 1 State Key Laboratory of Integrated Optoelectronics, College of Electronic Science and Engineering, Jilin University, 130012 Changchun, China; 2 Key Laboratory of New Energy and Rare Earth Resource Utilization of State Ethnic Affairs Commission, School of Physics and Materials Engineering, Dalian Minzu University, Dalian, 116600 China; 3 Department of Applied Physics, KTH Royal Institute of Technology, SE-106 91 Stockholm, Sweden

**Keywords:** Nanoparticles, Fluorescence resonance energy transfer

## Abstract

Exploring lanthanide light upconversion (UC) has emerged as a promising strategy to enhance the near-infrared (NIR) responsive region of silicon solar cells (SSCs). However, its practical application under normal sunlight conditions has been hindered by the narrow NIR excitation bandwidth and the low UC efficiency of conventional materials. Here, we report the design of an efficient multiband UC system based on Ln^3+^/Yb^3+^-doped core-shell upconversion nanoparticles (Ln/Yb-UCNPs, Ln^3+ ^= Ho^3+^, Er^3+^, Tm^3+^). In our design, Ln^3+^ ions are incorporated into distinct layers of Ln/Yb-UCNPs to function as near-infrared (NIR) absorbers across different spectral ranges. This design achieves broad multiband absorption withtin the 1100 to 2200 nm range, with an aggregated bandwidth of ~500 nm. We have identified a synthetic electron pumping (SEP) effect involving Yb^3+^ ions, facilitated by the synergistic interplay of energy transfer and cross-relaxation between Yb^3+^ and other ions Ln^3+^ (Ho^3+^, Er^3+^, Tm^3+^). This SEP effect enhances the UC efficiency of the nanomaterials by effectively transferring electrons from the low-excited states of Ln^3+^ to the excited state of Yb^3+^, resulting in intense Yb^3+^ luminescence at ~980 nm within the optimal response region for SSCs, thus markedly improving their overall performance. The SSCs integrated with Ln/Yb-UCNPs with multiband excitation demonstrate the largest reported NIR response range up to 2200 nm, while enabling the highest improvement in absolute photovoltaic efficiency reported, with an increase of 0.87% (resulting in a total efficiency of 19.37%) under standard AM 1.5 G irradiation. Our work tackles the bottlenecks in UCNP-coupled SSCs and introduces a viable approach to extend the NIR response of SSCs.

## Introduction

Given the increasing demand for energy, the development of clean and inexhaustible solar energy technologies promises significant longer-term benefits^1–3^. Silicon solar cells (SSCs), currently the most prevalent photovoltaic (PV) technology on the market, can continuously convert solar energy into electric energy. They are expected to maintain their dominance in the PV market for decades to come^4–9^. As their preparation technologies have matured, the power conversion efficiency of SSCs has exceeded 26.7%, leaving minimal room for further technical improvement^6,8^. Nevertheless, the ongoing quest to improve efficiency and reduce costs remains a critical objective. A significant barrier to further efficiency gains in SSCs is the intrinsic bandgap of silicon, which results in suboptimal solar spectrum utilization^10–12^. The inability to absorb photons with energy below the bandgap, along with the thermalization of photons with energies above the bandgap, leads to a loss of ~50% of the incident solar energy^10^. Thus, there remains a considerable gap to the theoretical limit^5,8,13^.

Currently, there are two strategies for improving the energy harvesting of SSCs, involving the technologies of tandem cells and luminescent conversion cells^11,12,14–24^. Tandem cells are mainly represented by perovskite/silicon cells, and great progress has been made^25–28^. Next to tandem cells, luminescent conversion cells, combining a spectrum conversion layer with SSCs, have particular utilities and merits, such as excellent compatibility to almost any types of current PVs, simple preparation process, low cost, and good stability^11,12^. Various luminescent conversion materials have been developed to enhance ultraviolet-blue or NIR responses of SSCs employing spectral down-shifting (and quantum cutting) or upconversion technology^10,11,29^. Particularly, lanthanide doped upconversion materials can convert low-energy photons (e.g., NIR) into high-energy photons (e.g., visible), and have been extensively explored for NIR light harvesting for solar cells (SCs), along with their wide-spread applications in e.g., 3D display and lighting, biological imaging, NIR photodetection, and super-resolution microscopy^30–36^. UC has been considered as a potential strategy to expand the NIR energy harvesting of SSCs to achieve a maximum theoretical efficiency of 47.6% for SSCs^37^. However, current SSCs incorporating UCNPs are only able to exhibit weak performance improvements, far behind expectations^37^.

Some key challenges remain before SSCs operating under nonconcentrated sunlight conditions can be enhanced via lanthanide light upconversion. First challenge comes with the relatively narrow absorption bands of lanthanide ions, resulting in a non-adequate coverage of the NIR region (mainly >1100 nm) of the solar spectrum^38–40^. Although this can be overcome to some extent by integrating a series of lanthanide ions, each harvesting a specific region, the lanthanide UC approach is further restricted by a second challenge, the low generation rate of upconverted light due to the multiphoton upconversion process feature, especially at low excitation intensities like nonconcentrated sunlight irradiation^41–43^. The requirement of relatively high excitation rates before such solar upconversion could start to become efficient would require the use of sun followers and solar light concentrators to track and focus the directly incident beam of the sun, limiting the practical applications^44,45^.

Based on these motivations, we have developed an efficient approach for collecting more of the NIR energy in sunlight by SSCs, by integration of multiband NIR responsive core-shell UCNPs. The UCNPs consist of multilayer Ln/Yb-UCNPs (Ln = Ho^3+^, Er^3+^, or Tm^3+^), in which Ln^3+^ ions are distributed in different layers, employed as NIR absorbers for capturing NIR energy in a broad range of 1100–2200 nm. More critically, we find that Yb^3+^ serves as a highly efficient electron pump, in synergistic action with the long-wavelength excitation NIR light, and simultaneously acts as a two-photon UC emitter. The synergistic effect that we have identified, termed the synthetic electron pump (SEP) effect, significantly mitigates energy losses due to downshifted emission and suppresses low-efficiency multiphoton UC processes. This leads to a substantial improvement in the UC efficiency of UCNPs, even under low light conditions. Through the development of nanoparticles, SSCs coated with optimized Ln/Yb-UCNPs demonstrate the broadest NIR response, extending up to 2200 nm. Notably, we achieved a remarkable enhancement in the absolute photon conversion efficiency (PCE) of 0.87% (on top of 18.5%) under standard AM 1.5G irradiation.

## Results

**Fig. 1:**
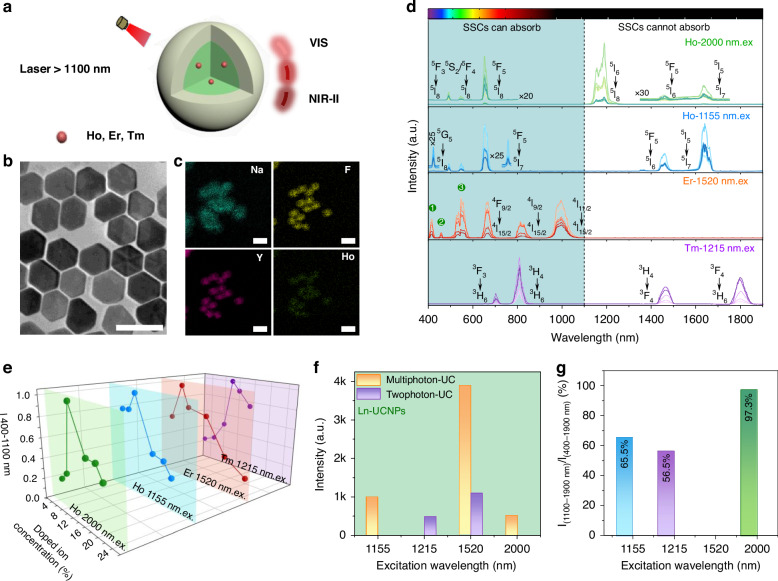
Availability analysis of Ln-UCNPs in the field of SSCs. **a** Schematic representation of multiband NIR responsive in Ln^3+^ doped UCNPs (NaYF_4_: Ln@NaYF_4_; Ln = Ho^3+^, Er^3+^, or Tm^3+^). **b**, **c** TEM and element mapping images of Ho-UCNPs with all scale bar 50 nm. **d** UC and down shifting emission spectra of Ln-UCNPs under NIR irradiation. The color of the spectral curve from light to dark symbolizes the dopant ion concentration from low to high. The processes ①, ②, and ③ represent the energy transition processes of Er^3+ 2^H_9/2_-^4^I_15/2_, ^4^F_3/2_/^4^F_5/2_-^4^I_15/2_, and ^2^H_11/2_/^4^S_3/2_-^4^I_15/2_, respectively. The used excitation power densities were 9.7 W cm^−2^, 10.1 W cm^−2^, 8.2 W cm^−2^ and 4.3 W cm^−2^ for 1155 nm, 1215 nm, 1520 nm, and 2000 nm, respectively. **e** Integral UC emission intensities (400–1100 nm) of Ln-UCNPs excited at different NIR wavelengths as a function of doping concentration. **f** Histograms of multiphoton and two photon UC emission intensities in Ln-UCNPs. **g** Integral emission ratios of 1100–1900 nm and 400–1900 in Ln-UCNPs under different excitation wavelengths

Firstly, NIR responsive core-shell UCNPs (NaYF_4_: Ln@NaYF_4_; Ln = Ho^3+^, Er^3+^, or Tm^3+^) were synthesized via the solvothermal method, as illustrated in Fig.1a^33,34^. High-resolution transmission electron microscopy (HR-TEM) images with element mapping analysis and X-ray diffraction (XRD) demonstrate the successful construction of the core-shell structure of UCNPs with a hexagonal phase and a diameter of ~35 nm (Fig.1b, c and Figs. S1, 2). An inert NaYF_4_ shell was included to minimize the surface defects of UCNPs and to protect the optically active core from quenching environments^33,46^. Figure 1d shows the full-spectral emission ranging of 400–1900 nm for three groups of Ln-UCNPs when pumped with NIR light at different wavelengths (2000 nm, 1155 nm, 1520 nm, and 1215 nm). The UC emission peaks of ^5^F_3_-^5^I_8,_^5^S_2_/^5^F_4_-^5^I_8_, ^5^F_5_-^5^I_8_, ^5^I_6_-^5^I_8_, ^5^F_5_-^5^I_6_, ^5^I_5_-^5^I_7_ (400-1700 nm) of Ho^3+^ under 2000 nm excitation, and the similar UC emission peaks of ^5^G_5_-^5^I_8,_^5^F_3_-^5^I_8,_^5^S_2_/^5^F_4_-^5^I_8_, ^5^F_5_-^5^I_7_ (400–800 nm) and down shifting emission bands of ^5^F_5_-^5^I_6_, ^5^I_5_-^5^I_7_ (1300–1700 nm) of Ho^3+^ under 1155 nm excitation were identified. The excitation spectra of Ho-UCNPs monitoring the UC visible emission in Fig. S3a further confirms that the effective pumping from two excitation bands in the range of 1100–1310 nm and 1935–2050 nm originate from the absorption of Ho^3+^ (^5^I_8_-^5^I_7_ and ^5^I_8_-^5^I_6_). For Er-UCNPs, the typical UC emission bands of Er^3+^ (^2^H_9/2_-^4^I_15/2_, ^4^F_3/2_/^4^F_5/2_-^4^I_15/2_,^2^H_11/2_/^4^S_3/2_-^4^I_15/2_, ^4^F_9/2_-^4^I_15/2_, ^4^I_9/2_-^4^I_15/2_, and ^4^I_11/2_-^4^I_15/2_ within 400–1100 nm) were obtained under 1520 nm excitation. In addition, the UC emission bands of ^3^F_3_-^3^H_6_ and ^3^H_4_-^3^H_6_ (400–900 nm) and down shifting bands of ^3^H_4_-^3^F_4_ and ^3^F_4_-^3^H_6_ (1300–1900 nm) were observed in Tm-UCNPs under 1215 nm excitation. The obvious excitation bands within 1405–1570 nm and 1160–1280 nm were recorded in Er-UCNPs and Tm-UCNPs, respectively (Fig. S3b, c). The involved transition processes are proposed in Fig. S4, based on literature reports^42,47–50^. These pumping bands for UCNPs, spanning from 1155 nm to 2000 nm, can provide a multiband NIR response possibility for SSCs. Figure 1e displays the integral emission intensity within 400–1100 nm (absorbable by SSCs) for these UCNPs as a function of Ln^3+^ concentration. Obviously, the emission intensities for different bands initially increase with increasing ion concentrations, and then reach a maximum at a certain concentration, and then start to decrease with further increasing the doping level. The optimum doping concentration is 10% for Ho^3+^, 8% for Er^3+^, and 10% for Tm^3+^, respectively.

These UCNPs can convert the otherwise unusable multiband NIR light so that it falls within the response region of SSCs from 400 to 1100 nm. However, some features may indicate low UC efficiency. Firstly, multiphoton UC emission (≥3 three photons) within 400–1100 nm tend to dominate under multiple wavelength excitations, as analyzed in Fig. 1f. These results are associated with the long excitation wavelengths used and the energy level structures of the involved ions. Multiphoton UC emissions would typically have much lower UC efficiency than two-photon UC emissions. Secondly, a lot of solar energy is still wasted with photon energies falling out of the bandgap of SSCs (>1100 nm) (Fig. 1d). Figure 1g shows the integrated emission intensity ratios between I_(1100–1900 nm)_/I_(400–1900 nm)_. Unfortunately, this wasted energy occupies a very high percentage of the total emission, 65.5%, 56.5%, and 97.3% under 1155 nm, 1215 nm, and 2000 nm, respectively. These results predict the low utility of these UCNPs in improving the performance of SSCs. This could explain why the use of UC for SSCs has this far been unsatisfactory.

**Fig. 2:**
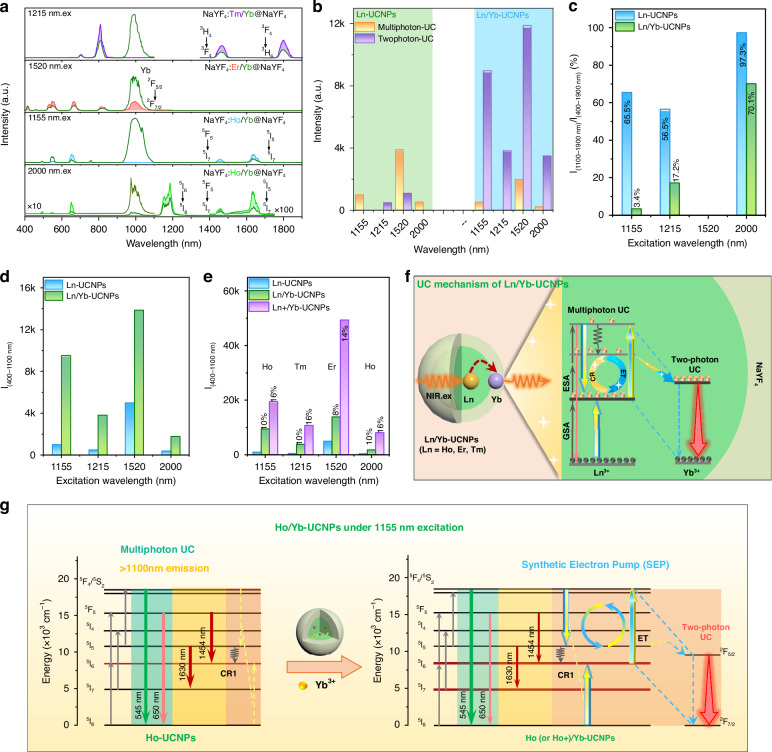
Proposed SEP mechanism and analysis of Ln/Yb-UCNPs availability in the field of SSCs. **a** Comparison of UC and down shifting emission spectra of NIR-excited Ln/Yb-UCNPs and Ln-UCNPs. The green curves are the spectra of Ln/Yb-UCNPs. **b** Histograms of multiphoton and two-photon UC emission intensities in Ln-UCNPs and Ln/Yb-UCNPs. **c** Integral emission ratio of 1100–1900 nm and 400–1900 nm in Ln-UCNPs and Ln/Yb-UCNPs. **d** Integral UC emission intensities of Ln-UCNPs and Ln/Yb-UCNPs within 400–1100 nm. **e** UC emission intensity of 400–1100 nm after the continued addition of Ln^3+^ to Ln/Yb-UCNPs, Ln^3+^ represents the continued doping of Ln^3+^ ion based on the previous optimized concentration. **f** Simplified model and overview of the mechanism of energy transfer processes in Ln/Yb-UCNPs. **g** Detailed description of the UC enhancement mechanism of Ho/Yb-UCNPs

We then sought to overcome this bottleneck problem through designing an emission energy acceptor using Yb^3+^ as a co-dopant in the UCNPs described above. Figure 2a displays the emission spectra of UCNPs (NaYF_4_: Ln, Yb^3+^@NaYF_4_; Ln = Ho^3+^, Er^3+^, or Tm^3+^) under different wavelength excitations. Surprisingly, strong emission located around 980 nm from Yb^3+^ (^2^F_5/2_-^2^F_7/2_) was clearly observed for all the samples, accompanied by decreased emission intensity both in the 400–900 nm and 1100–1900 nm regions. In Ho-UCNPs, we also attempted other ions as co-dopants (e.g., Er^3+^ and Tm^3+^), and found that Yb^3+^ performed best in suppressing the intensity of short-wavelength (<900 nm) UC and long-wavelength (>1100 nm) downshifting emissions of the nanoparticles (Fig. S5).

We then did more quantitative analyses on the fractions of the intensities of multiphoton UC and downshifting emissions in the total emission intensity. As shown in Fig. 2b and Figs. S6–8, the two-photon Yb^3+^ UC emission becomes dominant in Ln/Yb-UNCPs. Meanwhile, the integral emission intensity ratios between I_(1100–1900 nm)_/I_(400–1900 nm)_ significantly decrease to 3.4%, 17.2%, and 70.1% under 1155 nm, 1215 nm, and 2000 nm excitations, respectively, by optimizing the Yb^3+^ concentration (Fig. 2c and Fig. S9). Owing to the effective inhibition of low-efficiency multiphoton UC emission and the NIR emission out of the responsive region of SSCs, the overall UC emission intensity of I_(400–1100 nm)_ is remarkably enhanced for all the excitation bands, by a factor of 9.5, 7.8, 2.8, and 4.7, for 1155 nm, 1215 nm, 1520 nm, and 2000 nm excitation, respectively (Fig. 2d). In addition, it is also surprisingly found that the co-doping of Yb^3+^ allows for higher doping levels of the Ln^3+^ sensitizer ions without inducing concentration quenching (Fig. 2e). The increased optimal doping concentrations of Ln^3+^ increases the light absorption capacity of the nanoparticles and further boosts the UC emission. Compared to the initial Ln-UCNPs, the UC emission within the responsive region of SSCs of Ho+/Yb-UCNPs, Er+/Yb-UCNPs and Tm+/Yb-UCNPs under excitation wavelengths of 1155 nm, 1215 nm, 1520 nm, and 2000 nm can be maximally enhanced by a factor of 19.5, 22.1, 9.9, and 21.1, respectively (Fig. 2e and Fig. S10-11).

The astonishing performance of Yb^3+^ co-doping prompted us to decipher the underlying mechanism. We started with the following considerations. In the absence of Yb^3+^ ions, different Ln-UCNPs undergo specific UC pathways under different wavelengths excitation, as summarized in Fig. S4, and eventually generate multiple UC emission bands (<1100 nm). Note that these UC pathways, involving multiple steps, are always initiated by a ground state absorption (GSA) process induced by the excitation light and are then followed by excited state absorption (ESA) and cross-relaxation (CR) processes that particularly prevail at high doping concentrations. The greatly enhanced UC emission (<1100 nm) intensity after the addition of Yb^3+^ ions strongly indicates the optimization of certain step(s) in the overall multi-step UC emission process. We realize that the relaying ESA steps (after the GSA step) in the Ln-UCNPs are often off-resonant with the excitation light. Inspired by the generally reduced downshifting emission intensity, we speculate that the increased energy flow into the UC emitting channel originates from the promotion of the relaying steps after the initial GSA step. To be more specific, the originally dominant ESA steps are replaced by more efficient energy transfer (ET) steps from excited Yb^3+^ ions (initially activated by certain excited states of Ln^3+^) to Ln^3+^ at lower excited states. Subsequent CR processes, involving higher excited states of Ln^3+^ and their ground state, repopulate the lower excited states of Ln^3+^. These steps are continuously repeated, ultimately forming a positive feedback loop. In this process, Yb^3+^ ions continuously receive energy from Ln^3+^, with a portion contributing to the positive feedback loop and another part used for their own luminescence. Such a role of Yb^3+^ is schematically illustrated in Fig. 2f. It can be phenomenally formulated as a SEP effect, i.e., emptying electrons of Ln^3+^ at low excited states and concentrating them on the Yb^3+^ state.

As an example, we take the Ho/Yb-UCNPs under 1155 nm excitation of this SEP, illustrated in Fig. 2g. Briefly, in the absence of Yb^3+^ doping, the UC green and red emissions in Ho-UCNPs originate from a three- or two-photon UC process, starting from a GSA process (^5^I_8_ → ^5^I_6_, Fig. 2g). Meanwhile, there is substantial emission at 1454 nm and 1630 nm in the non-responsive region of SSCs (Fig. 1d, Fig. 2a). After doping Ho-UCNPs with suitable concentrations of Yb^3+^, these UC pathways can initially still take place. After a large enough population of Yb^3+^ at the excited state (via the Ho^3+ 5^I_5_ state), an ET process from Yb^3+^ to Ho^3+^ (i.e., ^2^F_5/2_ + ^5^I_6_ → ^5^F_4_/^5^S_2_ + ^2^F_7/2_) starts to take over as the dominant second critical step in the UC process. This would significantly change the dominant UC pathway and more efficiently increase the population of the Ho^3+ 5^F_4_/^5^S_2_ state by depopulating the ^5^I_6_ state. The subsequent CR process (e.g.,CR1 in Fig. 2g)^51,52^ together with a multiphonon decay process would eventually repopulate the Ho^3+ 5^I_6_ state. Such a loop efficiently inhibits multiphoton UC and downshifted emission from Ho^3+^. In the meantime, Yb^3+^ ions constantly receive energy from the Ho^3+ 5^I_5_ state and emit light at ~980 nm. The described mechanism is well supported by the experimental data. Besides the abovementioned much reduced intensity of the downshifted emission intensity and the strong Yb^3+^ emission, the green UC emission of Ho^3+^ is also found to be significantly increased in the presence of Yb^3+^, supporting our hypothesis of an increased importance of an ET process from Yb^3+^ to Ho^3+^ (i.e., ^2^F_5/2_ + ^5^I_6_ → ^5^F_4_/^5^S_2_ + ^2^F_7/2_) in the UC processes (Fig. 2a). In addition, the observed higher concentration quenching threshold of Ho^3+^ in the presence of Yb^3+^ also supports this mechanism, with the promoted CR process at elevated Ho^3+^ concentrations constructively contributing to the positive feedback loop. The augmented UC emission (<1100 nm) in Ho/Yb-UCNPs, Er/Yb-UCNPs, and Tm/Yb-UCNPs under 2000 nm, 1520 nm, and 1215 nm excitation can also be well explained by a similar SEP effect, see details in Fig. S12.

**Fig. 3:**
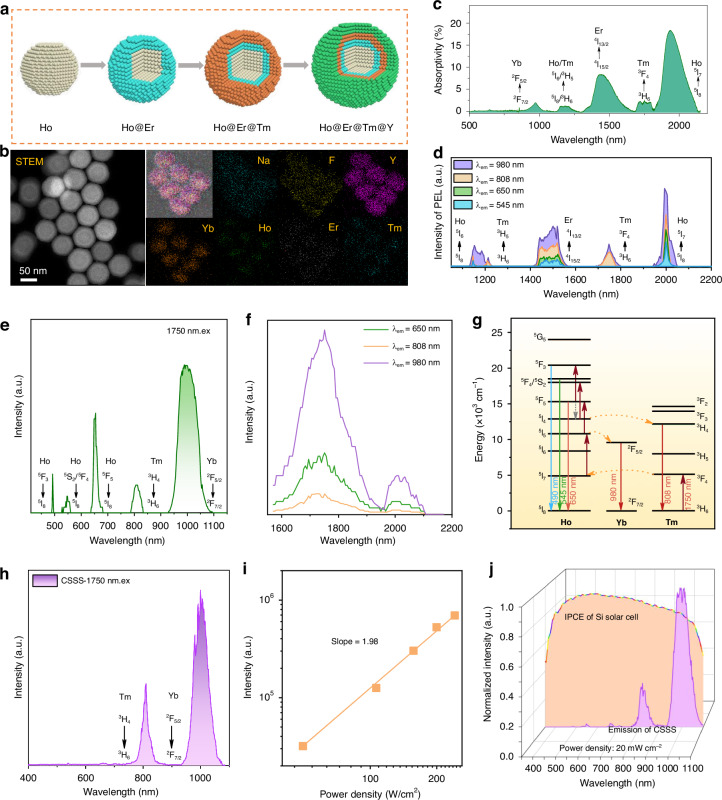
Structural characterization of CSSS and analysis of its optical properties. **a**, **b** Schematic diagram, HAADF characterization and elemental mapping images of CSSS samples. **c** Absorption spectrum of the CSSS film (with a thickness ~180 nm) ranging from 500 nm to 2200 nm. **d** Excitation spectra of CSSS samples correspond to different emission wavelengths. **e** UC emission spectrum of NaYF_4_: 4.5%Tm, 0.5%Ho, 5% Yb with an excitation wavelength of 1750 nm. **f** Excitation spectra of NaYF_4_: Tm, Ho, Yb samples. **g** Proposed energy level transition process of NaYF_4_: Tm, Ho Yb at the excitation wavelength of 1750 nm. **h** Emission spectrum of CSSS samples when excited at 1750 nm. **i** Power-density dependence of the UC emission (400–1100 nm) of CSSS nanoparticles under excitation of a broadband NIR light source (1100–2400 nm). **j** IPCE curve of SSC and the UC emission spectrum of the CSSS nanoparticles under broadband NIR excitation (1100–2400 nm). The excitation optical power density is 20 mW cm^−2^, which is similar to the intensity of near-infrared light in the solar spectrum ranging from 1100 to 2500 nm

Next, we integrated Ho^3+^, Er^3+^, and Tm^3+^-sensitized Ln/Yb-UCNPs into a unified system, to construct multiband NIR responsive UCNPs for SSCs, as displayed in Fig. 3a. The doping concentrations in different layers were inherited from the optimized samples in Fig. 2e. After optimization, the structure was settled to be NaYF_4_: 15%Yb, 16%Ho@NaYF_4_: 20%Yb, 14%Er@NaYF_4_: 20%Yb, 16%Tm@NaYF_4_ (CSSS, Fig. 3a). By the step-wise coating process, the particle size gradually increased from 27 nm to 59 nm (CSSS, Fig. S13a). The HAADF and EDS mapping images clearly demonstrate that uniform and core-multishell UCNPs were successfully prepared (Fig. 3b). The X-ray diffraction (XRD) patterns further confirm that the as-prepared UCNPs are in a highly crystallined hexagonal phase (Fig. S13b). The multilayer core-shell UCNPs exhibit exceptional multi-band absorption in the near-infrared region (Fig. 3c). Extraordinarily strong NIR absorption bands can be observed for the transitions of Tm^3+^, Ho^3+^, and Er^3+^, which are about one order of magnitude higher than the transitions in the 400–1100 nm range. Notably, the absorption peaks for the ^5^I_8_ → ^5^I_7_ transition of Ho^3+^ and the ^4^I_15/2_ → ^4^I_13/2_ transition of Er^3+^ are the most pronounced, with absorptivity ranging from 8% to 20%. Based on the absorption rate and doping concentration, we can determine that the absorption cross sections for the Er^3+^ ion’s ^4^I_15/2_ → ^4^I_13/2_ transition and the Ho^3+^ ion’s ^5^I_8_ → ^5^I_7_ transition are $2.6\times {10}^{-18}\,{{cm}}^{2}$ and $4.9\times {10}^{-18}\,{{cm}}^{2}$, respectively. These values significantly surpass those documented in earlier studies, which typically ranged around 10^−21 ^cm^2^ (Supplementary Note 1). To verify the multiband NIR responsive ability, the excitation spectra of the CSSS nanoparticles were measured by monitoring the 980 nm (Yb^3+^), 808 nm (Tm^3+^), 650 nm (Ho^3+^), and 545 nm (Er^3+^) emissions (Fig. 3d). The multiband NIR response including 1120–1235 nm (^5^I_8_-^5^I_6_ for Ho^3+^, ^3^H_6_-^3^H_5_ for Tm^3+^), 1405–1570 nm (^4^I_15/2_-^4^I_13/2_ for Er^3+^), 1690–1800 nm (^3^H_6_-^3^F_4_ for Tm^3+^), and 1935–2050 nm (^5^I_8_-^5^I_7_ for Ho^3+^) were identified, achieving a wavelength coverage of ~500 nm in the >1100 nm region (Fig. 3d). Compared with previous UC systems (Supplementary Table S2), an unprecedented increase in excitation spectrum coverage is obtained, which is of great significance for the energy utilization of infrared light.

Intriguingly, a new excitation band peaked at 1750 nm was also observed when monitoring the 980 nm (Yb^3+^) and 808 nm (Tm^3+^) emissions (Fig. 3d), which was assigned to the GSA transition of Tm^3+^ (^3^H_6_ → ^3^F_4_). It should be noted that UC emission was not directly observable in Tm-UCNPs or Tm/Yb-UCNPs under 1750 nm excitation. After analysis, we hypothesize that this new excitation band is assisted by the involvement of Ho^3+^ in its excited state, which can overcome the energy mismatching in the UC process. A strong indication comes from the observation that the 2000 nm excitation band for Ho^3+^ can also generate UC emission of Tm^3+^ (808 nm) in the CSSS nanoparticles but not in Tm-UCNPs or Tm/Yb-UCNPs. Cation intermixing often happens during the synthesis of core-shell UCNPs (Fig. S14)^53^. To further support our hypothesis, we synthesized NaYF_4_: 4.5% Tm, 0.5% Ho, 5% Yb homogeneously doped UCNPs and recorded their UC emission and excitation spectra. The UC emission bands from Ho^3+^ and Tm^3+^ were clearly observed under 1750 nm excitation (Fig. 3e). Then, similar excitation bands centered at 1750 nm and 2000 nm, assigned to the GSA of Tm^3+^ (^3^H_6_-^3^F_4_) and Ho^3+^ (^5^I_8_-^5^I_7_), respectively, were also observed when monitoring the UC emission of Ho^3+^ (650 nm) and Tm^3+^ (808 nm), as shown in Fig. 3f. It can be concluded that bidirectional energy transfer between Ho^3+^ and Tm^3+^ and their synergistic effect creates a new excitation band of 1690–1800 nm for the CSSS nanoparticles. Possible energy transfer pathways are illustrated in Fig. 3g. Briefly, when irradiated at 1750 nm, the ground state Tm^3+^ ions were pumped to the ^3^F_4_ energy level, followed by energy transfer to Ho^3+^ ions to populate their ^5^I_7_ energy state. Higher Ho^3+^ excited states can be subsequently populated, followed by the repopulation of Tm^3+^ and Yb^3+^ excited states. Eventually, bright 808 nm (Tm^3+^) and 980 nm (Yb^3+^) UC emission were obtained in the CSSS nanoparticles through a two-photon UC process (Fig. 3h).

The UC emission spectra of CSSS UCNPs was recorded under the excitation of a NIR light source with a cutting off wavelength at around 1100 nm (Fig. S15). A set of UC emission (400–1100 nm) spectra were measured with varying excitation power densities (Fig. S16). The integrated UC intensities are plotted against the excitation power densities in Fig. 3i. The linear fit of the data yielded a slope efficiency of 1.98, indicating the predominance of a two-photon UC process. As shown in Fig. 3j, the UC emission bands obtained from Yb^3+^, Tm^3+^, Ho^3+^, and Er^3+^ conform well with the incident photon-to-electron conversion efficiency (IPCE) curves of SSCs with excellent coverage.

**Fig. 4:**
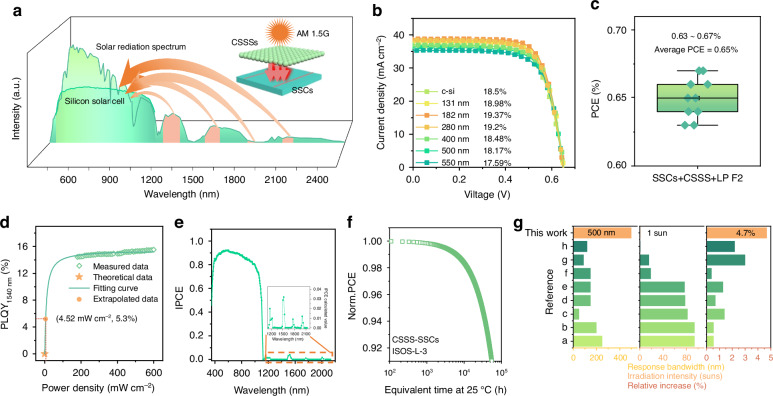
Effectiveness and breakthrough of CSSS application in SSCs. **a** Schematic diagram of the responsive range in SSCs combined with CSSS. The inset depicts a schematic model of the CSSS-coated SSC surface with the light source being AM 1.5 G. **b** I–V curves of SSC coated with CSSS films of different thicknesses. **c** Statistical tests on PCE of SSCs coated with CSSS films. Under AM 1.5G standard sunlight filtered by a lowpass filter (LP F2) with a cutoff wavelength of 1100 nm. **d** The measured upconversion PLQY of CSSS under 1520 nm excitation with different power densities and extrapolated PLQY at low power density using the formula $\eta =\frac{{\eta }_{s}\times \frac{\rho }{{\rho }_{b}}}{1+\frac{\rho }{{\rho }_{b}}}$. **e** IPCE curves of SSCs coated with CSSS film. The inset shows an enlarged 1100–2200 nm IPCE curve graph. **f** Normalized PCE of CSSS coated SSC with equivalent aging time at ISOS-L-3. **g** Summarized response bandwidth, irradiation intensity, and performance increase of combining UC conversion layer with SSCs, corresponding to Supplementary Table 3

We then applied the optimized CSSS UCNPs as spectrum conversion materials to broaden the spectral response range of SSCs, as illustrated in Fig. 4a. Firstly, CSSSs were self-assembled on the surface of commercial single crystal SSCs (area: 150 mm$\times$150 mm) to form a thin film. By adjusting the parameters involved in the liquid-phase deposition protocol, different film thicknesses can be produced, ranging from 130 to 550 nm (Fig. S17). According to the AFM characterization, the resultant CSSSs films were relatively flat, with low roughness of 6.19 nm, which is beneficial to reduce the light scattering effect of this layer (Fig. S18). Figure 4b shows the I-V curves of the SSCs coated with different-thickness CSSS films. In comparison to the uncoated SSCs with a PCE of 18.5% achieved, the PCE of the SSCs coated with a CSSS film of 182 nm was increased to 19.37% (a net increase of 0.87% and 2 mA cm^−2^ for short-circuit current). The increase of PCEs for SSCs is dominated by the short-circuit currents. Further increasing the film thicknesses, the PCE gradually decreases. Meanwhile, the performance of SSCs coated with Ln/Yb-UCNPs (Ln = Ho^3+^, Er^3+^, Tm^3+^) were also performed, demonstrating the improvement of PCEs (SFig. S19). The repeatability of the SSCs with or without CSSS films was confirmed on different positions of the same SSC and on different devices (Fig. S20). The results from both the diffuse reflectance and transmission spectra show that CSSS films of suitable thicknesses have minimal light losses (Fig. S21). This supports the idea of using CSSS as a light conversion layer to enhance the photovoltaic efficiency of SSCs. As a contrast, we tested the I-V curves of SSCs coated with undoped NaYF_4_ (size = 59.6 nm) as a function of different thicknesses (Fig. S22). Apparently, the PCEs of SSCs did not increase but decreased, indicating that the multiband NIR light conversion of CSSS boosts the performance of SSC. To further clarify the contribution of multiband NIR responsive UC, we measured the PCE of CSSS-coated SSCs under AM 1.5 G with the assistance of a low-pass filter with a cutoff wavelength of 1100 nm (Fig. S15b). As can be seen from Fig. 4c, the average PCE of SSCs reaches 0.65% induced by lanthanide ions upconversion pathway of CSSSs. The PCE mismatch of SSC coated with SSCs under AM 1.5 G with or without a low-pass filter <1100 nm may come from the down-shifting ability of CSSS to UV lights (Fig. S23). The improved PCE of CSSS-coated SSCs stems from the exceptional upconversion capability of CSSS under standard solar irradiation. Therefore, we recorded CSSS upconversion photoluminescence quantum yields (PLQYs) across different excitation power densities to estimate their PLQYs at low power densities. For this estimation, the following fitting formula was used,

1 $$\eta =\frac{{\eta }_{s}\times \frac{\rho }{{\rho }_{b}}}{1+\frac{\rho }{{\rho }_{b}}}$$

where *ρ* denotes the excitation power density, and *ρ*_*b*_ the balancing power density, and *η*_*s*_ represents the maximum PLQY achieved at saturation excitation power density. In the fitting, *ρ*_*b*_ and *η*_*s*_ were the fitted parameters. This formula can provide a good estimate for the PLQY of two-photon upconversion luminescence at arbitrary excitation intensities^54^. Based on thePLQY for the CSSS under 1520 nm laser radiation, it is suggested that within the 1400–1650 nm range of the solar spectrum (with a power density of ~4.52 mW cm^−2^), CSSS achieves a PLQY about 5.3% (Fig. 4d). This corresponds to an estimated 0.67% improvement in PCE for CSSS-coated SSCs, consistent with the findings illustrated in Fig. 4c (Supplementary Note 2). Considering the limited detection range of IPCE measurements, the IPCE curve for the SSC with CSSS coating was calculated (Supplementary Note 3 and Supplementary Table S1). The spectral response range was expanded to 2200 nm, leading to the increase in the PCE of SSC (Fig. 4e). Furthermore, the meticulous testing and extrapolation reveal that CSSS-coated SSCs exhibit excellent stability originating from the outstanding stability of UCNPs. The PCE of CSSS-coated SSCs was monitored for 5000 h at 120 °C and 50% relative humidity to determine the T_90_ (the time until the PCE of the SSCs drops to 90% of its initial value). An acceleration factor (AF) of 11.02 at 120 °C was calculated based on the test results shown in Fig. S24, 25 and it can therefore be reasonably inferred that there is only a 10% PCE decay over 55,100 continuous hours at 25 °C and 50% relative humidity, conforming to ISOS-L-3 standards^55^ (Fig. 4f) (Supplementary Note 4). Compared with previous reports employing UC materials for SSC applications, we have achieved an unparalleled enhancement of PCEs with broad excitation bandwidths and under low irradiation intensities (Fig. 4g and Supplementary Table 3). In summary, we have obtained: (1) A substantial PCE increase by lanthanide upconversion for SSCs under nonconcentrated sunlight irradiation; (2) an unprecedented broad-band NIR response of SSCs, covering close to 500 nm; (3) Remarkable lanthanide UC emission efficiencies for long-wavelength (1100–2200 nm) NIR excitation by introducing Yb^3+^ as electron pump ions.

## Discussion

In this work, we fabricated Lanthanide-doped UCNPs (NaYF_4_: Ln@NaYF_4_; Ln = Ho^3+^, Er^3+^, or Tm^3+^) and observed UC emission and NIR downshifted emissions under multiband NIR excitation at 1155 nm, 1215 nm, 1520 nm, and 2000 nm. To boost the quantum efficiency of the long-wavelength NIR light-generated UC emission, we utilized Yb^3+^ ions as initiators of a synthetic electron-pumping effect. This effect results from the synergistic interplay of energy transfer and cross-relaxation between Yb^3+^ and other Ln^3+^ ions (Ho^3+^, Er^3+^, Tm^3+^), effectively depleting electrons from the lower excited states of Ln^3+^ to the excited state of Yb^3+^, resulting in strong Yb^3+^ luminescence (~980 nm) that aligns well with the spectral response of SSCs. Moreover, we constructed core-multishell UCNPs (denoted as CSSS, the main ingredient is NaYF_4_: 15%Yb, 16%Ho@NaYF_4_: 20%Yb, 14%Er@NaYF_4_: 20%Yb, 16%Tm@NaYF_4_) incorporating Ln^3+^ ions (Ho^3+^, Er^3+^, Tm^3+^) as multiband NIR absorbers and Yb^3+^ ions as two-photon UC emitters. Importantly, we discovered a new excitation band at 1750 nm, attributed to the bidirectional energy transfer between Ho^3+^ and Tm^3+^ ions. This optimized multilayer Ln/Yb-UCNPs (Ln = Ho^3+^, Er^3+^, or Tm^3+^) exhibit a broad multiband NIR response distributed across the range from 1100 to 2200 nm, with an aggregated bandwidth of ~500 nm. This design achieves an overall UC PLQY of 12% under standard solar irradiation. By coating SSCs with the multiband responsive Ln/Yb-CSSSs and through integration with the optimized UC nanomaterials, we extended their responsiveness up to 2200 nm, achieving a substantial increase in PCE of 0.87% under standard sunlight conditions. Furthermore, CSSS-coated SSCs demonstrated durability, maintaining functionality for more than 55,100 h under standard temperature and humidity conditions, making them highly valuable for practical applications.

## Materials and methods

### Materials

Octadecene (ODE, 90%), oleic acid (OA, 90%), HoCl_3_•6H_2_O (99.99%), YbCl_3_•6H_2_O (99.99%), ErCl_3_•6H_2_O (99.9%), YCl_3_•6H_2_O (99.9%), TmCl_3_•6H_2_O (99.9%), Sodium hydroxide (NaOH; >98%), ammonium fluoride (NH_4_F; >98%), oleic acid (OA, technical grade 90%, Aldrich), 1-octadecene (ODE, technical grade 90%, Aldrich), cyclohexane (>98%), were all purchased from Sigma‐Aldrich and used as starting materials without further purification.

### Synthesis of core-shell UCNPs

The designed NaYF_4_:Ho^3+^, Ln^3+^@NaYF_4_ (Ln = Er, Yb, Tm) UCNPs were synthesized using a reported solvothermal method. A 100 mL three-neck flask was charged with oleic acid (6 mL), octadecene (15 mL), and YCl_3_·6H_2_O, YbCl_3_·6H_2_O, HoCl_3_·6H_2_O, and LnCl_3_·6H_2_O in the appropriate molar ratio, totaling 2 mmol of lanthanides. The mixture was stirred at 140 °C for 1 h to form a lanthanide complex, then cooled to room temperature. NaOH (5 mmol) and NH_4_F (8 mmol) in methanol (6 mL) were added, and the mixture was stirred for 1 h, then heated at 300 °C for 1.5 h under nitrogen. After cooling, the UCNPs were collected and washed with ethanol-cyclohexane (1:1 v/v) and redispersed in 5 mL cyclohexane for shell growth.

For NaYF_4_: Ho^3+^, Ln^3+^@NaYF_4_ core-shell UCNPs, a shell precursor solution containing 2 mmol YCl_3_·6H_2_O was prepared similarly. After 45 min, the mixture was cooled, and NaOH (5 mmol) and NH_4_F (8 mmol) in methanol (6 mL) were added. The mixture was stirred at 25 °C for 15 min, heated to 80 °C for 30 min, then heated to 320 °C for 1.5 h. The core-shell UCNPs were collected and washed as described.

### Synthesis of core-shell-shell-shell (CSSS)

The multilayered core-shell nanocrystals were grown stepwise using the same method described above for the core-shell UCNPs.

### Fabrication of CSSS coated silicon solar cells

The UCNPs (0.1 mmol, 0.2 mmol, 0.3 mmol, 0.5 mmol, 0.6 mmol and 0.9 mmol) were dispersed in 10 mL hexane solution. The silicon solar cell was dropped vertically into the solution and placed in an oven at 30 °C for 4 h. With the slow volatilizing of hexane, the UCNPs were self-assembled on the surface of silicon solar cell.

### Characterization

The purities and phase structures of the products were characterized by X-ray diffraction. The morphologies of UCNPs films were examined using an Asylum MFP-3D atomic force microscope and recorded with a Hitachi H-8100IV TEM at 200 kV in EFTEM mode, using a 20 eV energy slit for HRTEM micrographs. For emission, luminescence kinetics, and excitation spectrum measurements, the samples were pumped using a laser system with a Nd: YAG laser (1064 nm), third-order Harmonic-Generator (355 nm), and a tunable optical parameter oscillator (OPO, Continuum Precision II 8000) with a 10 ns pulse duration, 10 Hz repetition frequency, and 4–7 cm^−1^ line width. A visible photomultiplier (350–850 nm or 950–1650 nm) combined with a double-grating monochromator was used for spectral collection. Solar cell devices were tested under AM 1.5G, 100 mW/cm² illumination with a Class A solar simulator (ABET Sun 2000), calibrated with a Silicon cell (RERA Solutions RR-1002), using a Keithley 2400 source meter for J-V measurements from +1.5 V to −1.5 V in ambient conditions.

## Supplementary information

Supplementary file for A multiband NIR upconversion core-shell design for enhanced light harvesting of silicon solar cells

## Data Availability

All data are true and reliable. Upon reasonable request, it may be obtained from the corresponding author.
