# Supplementary material for: A multiband NIR upconversion core-shell design for enhanced light harvesting of silicon solar cells
Source: Light Sci Appl. 2024 Nov 25;13:312. doi: 10.1038/s41377-024-01661-5 (PMC11586394; doi:10.1038/s41377-024-01661-5)
Supplement: Supplementary file 1 — Supplementary file for A multiband NIR upconversion core-shell design for enhanced light harvesting of silicon solar cells [file 41377_2024_1661_MOESM1_ESM.docx]

**Supplementary Information**

**A multiband NIR upconversion core-shell design for enhanced light harvesting of silicon solar cells**

Yue Wang^1^, Wen Xu^2*^**,** Haichun Liu^3^, Yuhan Jing^2^, Donglei Zhou^1^, Yanan Ji^2^, Jerker Widengren^3^, Xue Bai^1^, Hongwei Song^1*^

^1^ State Key Laboratory of Integrated Optoelectronics, College of Electronic Science and Engineering, Jilin University, 130012 Changchun, China

^2^ Key Laboratory of New Energy and Rare Earth Resource Utilization of State Ethnic Affairs Commission, School of Physics and Materials Engineering, Dalian Minzu University, Dalian, 116600, P.R. China

^3^ Department of Applied Physics, KTH Royal Institute of Technology, SE-106 91 Stockholm, Sweden

E-mail: [xuwen@dlnu.edu.cn, songhw@jlu.edu.cn;](mailto:%20xuwen@dlnu.edu.cn,%20songhw@jlu.edu.cn;)

**Supplementary Note 1:**

**Calculation of absorption cross sections for sensitizer Er^3+^ ions and Ho^3+^ ions in CSSS**

Returning to the properties of *β*-NaYF_4_, it exhibits a density of 4.3 g cm^-3^ and a molar weight of 188 g mol^-1^, resulting in a concentration of Y^3+^ atoms of 1.4 × 10^22^ cm^-3^. The concentration of Er^3+^, Ho^3+^ in CSSS is 14%, 16% respectively. The absorption cross section of Er^3+^ and Ho^3+^ can be calculated using the following equation:

$$\begin{aligned} \alpha=\sigma*N\#\left( AUTONUM \backslash* Arabic \right) \end{aligned}$$

where *α* is the absorption coefficient, *σ* is the absorption cross section of rare earth ions, and *N* is the atomic concentration of Er^3+^, Ho^3+^ in CSSS. The calculations show that the absorption cross section of Er^3+^ ions *σ_Er-1520 nm_=*2.6 × 10^-18^ cm², and for Ho^3+^ ions *σ_Ho-2000 nm_*=4.9 × 10^-18^ cm². These values notably exceed earlier research findings, which were around 10^-21^ cm². This provides a theoretical foundation for CSSS to exhibit relatively high quantum yields under standard solar irradiation conditions.

**Supplementary Note 2:**

**PLQY projection at low power and its PCE calculation**

We measured the change in upconversion quantum yield (PLQY) of CSSS under high power density at the 1520 nm wavelength in the solar spectrum. Subsequently, we used the dependenceof PLQY on excitation power densities to estimate CSSS's PLQY under low power density relevant to solar irradiation. The fitting equation used is as follows:

$$\begin{aligned} \eta=\frac{\eta_{s}\times\frac{\rho}{\rho_{b}}}{1+\frac{\rho}{\rho_{b}}}\#\left( AUTONUM \backslash* Arabic \right) \end{aligned}$$

where *ρ* denotes excitation power density and *ρ_b_* the balancing power density, and *η_s_* represents the maximum PLQY achieved at saturation excitation power density. The formula can provide a good estimate for PLQY of two-photon upconversion luminescence at arbitraty excitation power densities. The fitting results are shown in Fig. 4d in the main text.

The excitation spectrum of CSSS at 1520 nm has a power density of 4.52 mW cm^−2^ in the solar spectrum, with an estimated upconversion PLQY of approximately 5.3% from the fitted curve. The upconversion PLQY of CSSS films under 1155 nm, 1750 nm, and 2000 nm excitation bands in the solar spectrum can be determined based on the integral intensities of each band in the excitation spectrum.

$${PLQY}_{1155 nm}={PLQY}_{1520 nm}\times0.72=3.82\%$$

$${PLQY}_{1750 nm}={PLQY}_{1520 nm}\times0.32=1.7\%$$

$${PLQY}_{2000 nm}={PLQY}_{1520 nm}\times0.22=1.2\%$$

In short, CSSS films achieve a 12% upconversion PLQY under near-infrared light with wavelengths exceeding 1100 nm in the solar spectrum.

According to the energy conversion relationship it can be determined:

$$\begin{aligned} PCE=\frac{P_{output}}{P_{input}}\#\left( AUTONUM \backslash* Arabic \right) \end{aligned}$$

$$\begin{aligned} P_{output}=P_{total}\times17\%\times A\times\eta\times{IPCE}_{400-1100 nm}\#\left( AUTONUM \backslash* Arabic \right) \end{aligned}$$

$$\begin{aligned} P_{input}=P_{total}\times20\%\#\left( AUTONUM \backslash* Arabic \right) \end{aligned}$$

Where $P_{total}$ represents the complete radiant power density of the AM 1.5G standard solar spectrum; 17% indicates the percentage of power density attributed to the absorption wavelength coverage of the CSSS film within the AM 1.5G standard solar spectrum; while 20% reflects the percentage of power density beyond 1100 nm in the solar spectrum; *A* represents the average absorption rate of CSSS films, defaulted to 4.8%; and *IPCE_400-1100 nm_* denotes the photon-to-electron conversion efficiency of silicon solar cells in the range of 400-1100nm, estimated at about 88%. *η* represents the energy conversion efficiency of the CSSS film, calculated as $\eta=PLQY\times\frac{{h\omega}_{2}}{{h\omega}_{1}}$, where *PLQY* is the quantum yield of the CSSS film excited by light with wavelengths exceeding 1100 nm in the solar spectrum; *h* is the Planck constant; *ω_2_* primarily corresponds to a wavelength of 980 nm due to emitted light characteristics. The excitation spectrum spans four bands: 1120-1235 nm, 1405-1570 nm, 1690-1800 nm, and 1935-2050 nm. In this framework, the default wavelength for *ω_1_* is 1520 nm.

According to the fitting in Fig. 4d and Eq. (3)-(5), the PCE of CSSS-coated SSCs reached 0.67% under solar irradiation exceeding 1100 nm.

**Supplementary Note 3:**

**Test characterization of IPCE curves**

OPO (Continuum Precision II 8000) laser was used as the light source and the receiving device was Keithley 2400. OPO laser can output continuously tunable near infrared light. Several points within the coverage range of CSSS sample excitation wavelength were selected, and the corresponding photocurrent output was tested and recorded under dark conditions. IPCE calculation formula is as follows:

$$\begin{aligned} IPCE\left( \lambda\right)=\frac{I_{photo}\left( \lambda\right)}{P_{in}\left( \lambda\right)}\#\left( AUTONUM \backslash* Arabic \right) \end{aligned}$$

where $I_{photo}(\lambda)$ is the short-circuit current density generated by the SSC under monochromatic light irradiation of wavelength $\lambda$, $\lambda$ is the incident light wavelength, and $P_{in}(\lambda)$ is the power density of the incident light. The calculated values of all parameters and IPCE are shown in the table below.

Supplementary Table 1: The values of each parameter involved in the IPCE calculation.

| Incident Wavelength (nm) | Inci dent light intensity (mW cm^-2^) | Short-circuit current density (mA cm^-2^) | IPCE |
| --- | --- | --- | --- |
| 1140 | 32.9 | 0.11 | 0.0033 |
| 1160 | 32.17 | 0.64 | 0.02 |
| 1180 | 31.55 | 0.25 | 0.008 |
| 1200 | 31.28 | 0.28 | 0.009 |
| 1220 | 30.86 | 0.31 | 0.01 |
| 1480 | 56.81 | 0.08 | 0.0014 |
| 1500 | 55.77 | 1.62 | 0.029 |
| 1520 | 54.98 | 1.76 | 0.032 |
| 1540 | 53.5 | 0.75 | 0.014 |
| 1740 | 24.47 | 0.03 | 0.0013 |
| 1760 | 23.46 | 0.21 | 0.009 |
| 1785 | 22.85 | 0.73 | 0.032 |
| 1985 | 16.97 | 0.04 | 0.0021 |
| 2005 | 16.32 | 0.21 | 0.012 |
| 2025 | 15.41 | 0.08 | 0.0052 |

**Supplementary Note 4:**

**Stability calculations for CSSS wrapped SSCs**

A crucial factor that significantly affects the commercialization of solar devices is stability. The aging experiments were performed to evaluate the stability of CSSS-SSCs devices. We maintained a constant RH level of 50% and a constant light intensity of AM 1.5G (100 mW/cm^2^) throughout the experiment to ensure consistent conditions. The aging process of the devices was accelerated by subjecting them to different temperatures: 25 ℃, 80 ℃, and 120 ℃ (Fig. S24). This experimental setup adheres to the ISOS-L-3 standard. The CSSS-SSCs were operated at 25 ℃ for approximately 5,000 hours, showing PCE with almost no attenuation. When the temperature was further increased to 120℃, the PCE of SSC remained at 91% of the initial value after 5000 hours. This is due to the super stability of CSSS. The life acceleration factor (AF) for the accelerated aging process of the presumed device was calculated by the following equation:

$$\begin{aligned} AF=\frac{k_{acc}}{k_{ref}}=\exp\left( \frac{E_{a}}{k_{B}} \right)\left[ \frac{1}{T_{ref}}-\frac{1}{T_{acc}} \right]\#\left( AUTONUM \backslash* Arabic \right) \end{aligned}$$

where *k_B_* is the Boltzmann constant, *T_acc,_* and *T_ref_* are the operating temperatures during aging at accelerated and standard operating conditions, and the AF factor establishes a relationship between the life of the device under normal operating conditions and its life under high-stress conditions. *Ea* is the activation energy of degradation, which can be derived from the Arrhenius equation:

$$\begin{aligned} k\left( T \right)=Aexp\left( \frac{{-E}_{a}}{k_{B}T} \right)\#\left( AUTONUM \backslash* Arabic \right) \end{aligned}$$

where *k(T)* is a degradation rate at temperature *T*, and *A* is constant. The degradation rate as a function of inverse temperature is represented in Fig. S25. A single Arrhenius function describes the degradation rate across the entire temperature range, with the slope of this function indicating the *E_a_* value. The *E_a_* value calculates the AF factor for the CSSS-SSC. The operating time equivalent of the aging device at 25 °C is determined by multiplying the aging time by the AF, as shown in Fig. 4f. Based on the findings in Fig. S24, the average T_90_ of the CSSS-SSCs was 5000 hours at 120 ℃. The AF of the CSSS-SSCs is 11.02 at 120 ℃. We can estimate the T_90_ of CSSS-SSCs to be 55100 hours at 25 °C (Fig. 4f).

**Supplementary Figures and Tables:**


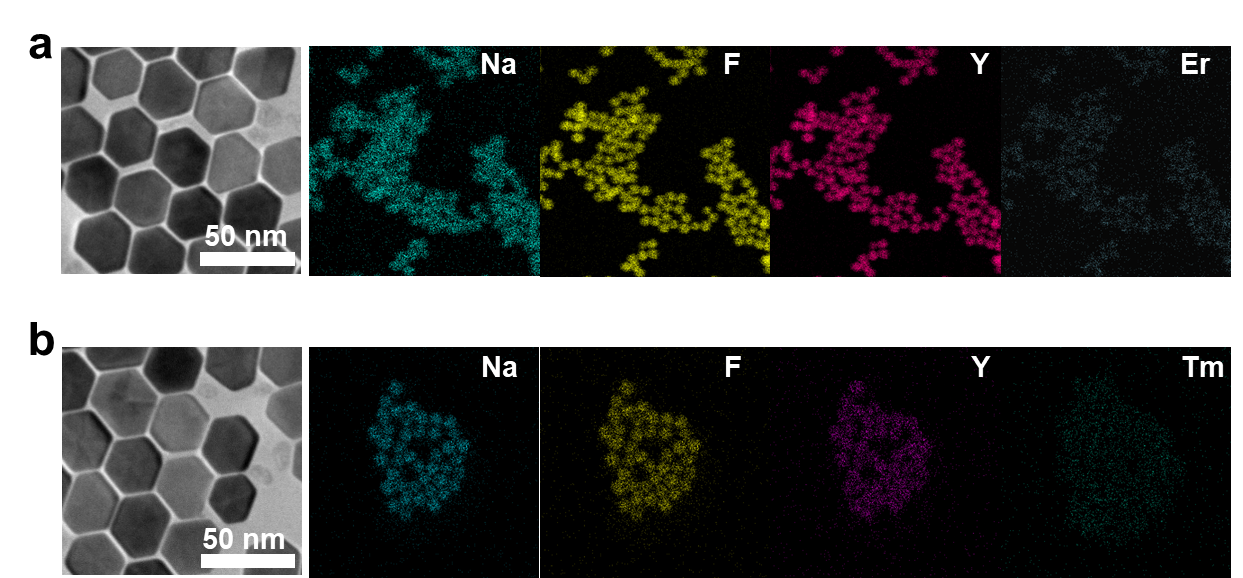
**Figure S1.** (a-b) TEM images and element mapping images of as-synthesized NaYF_4_: Er@NaYF_4_ and NaYF_4_: Tm@NaYF_4_.


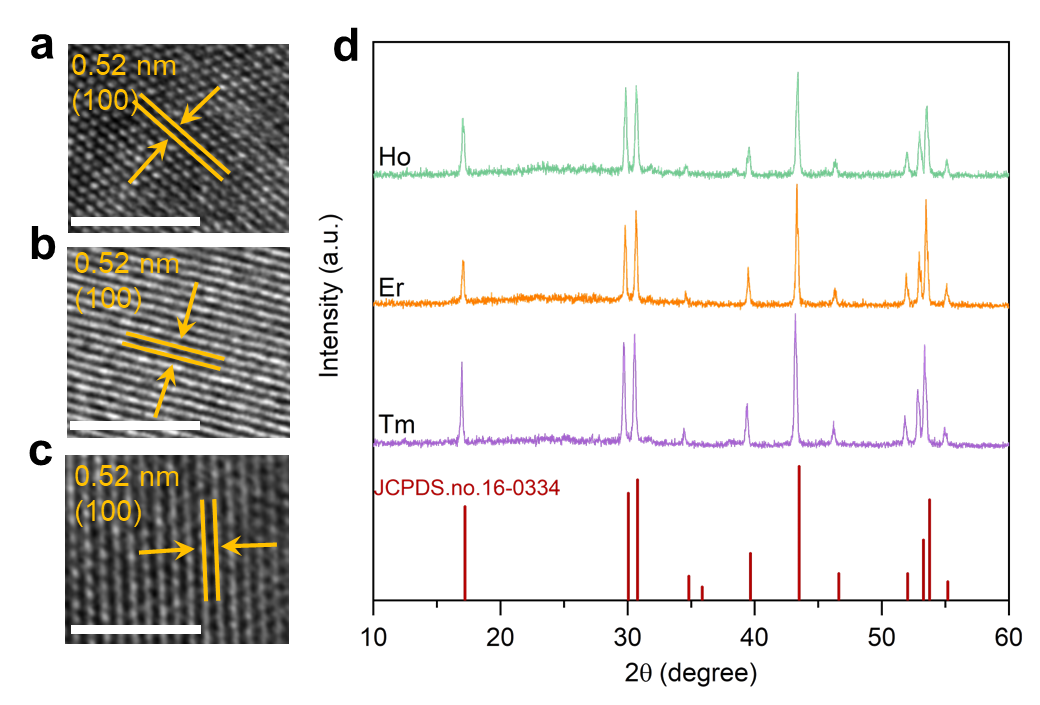
**Figure S2.** (a-c) High-resolution transmission electron microscopy images of as-synthesized NaYF_4_: Ho@NaYF_4_, NaYF_4_: Er@NaYF_4_, and NaYF_4_: Tm@NaYF_4_; the scale is 5 nm. (d) XRD diffraction patterns of as-synthesized NaYF_4_: Ho@NaYF_4_, NaYF_4_: Er@NaYF_4_, and NaYF_4_: Tm@NaYF_4_.


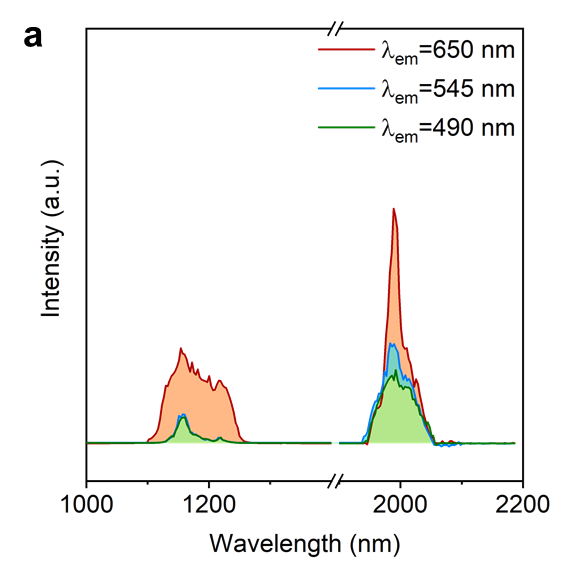


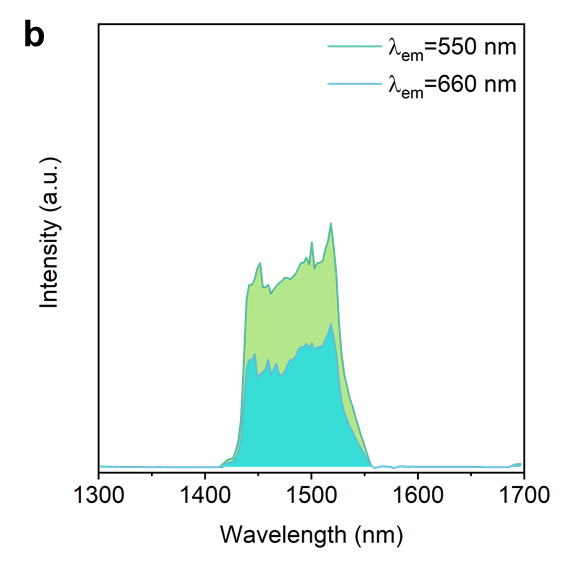


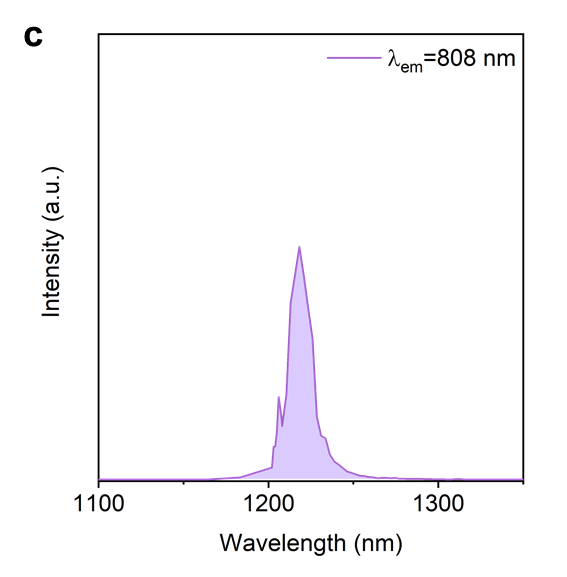


**Figure S3.** (a-c) Excitation Spectrum of as-synthesized NaYF_4_: Ho@NaYF_4_, NaYF_4_: Er@NaYF_4_, and NaYF_4_: Tm@NaYF_4_.


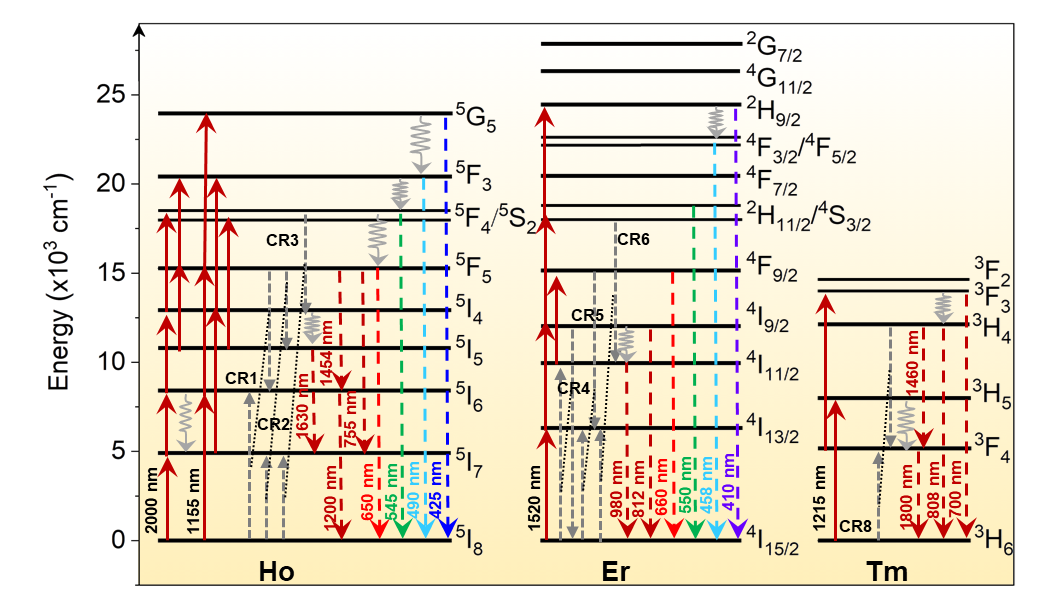
**Figure S4.** The energy level transition process of Ln-UCNPs under NIR is exciting. Ho-UCNPs respond to two NIR wavelengths, specifically at 1155 and 2000 nm. Under 1155 nm laser irradiation, electrons through a three-photon UC process, leading to visible emissions at 425 nm (^5^G_5_-^5^I_8_), 490 nm (^5^F_3_-^5^I_8_), 545 nm (^5^F_4_/^5^S_2_-^5^I_8_), and 650 nm (^5^F_5_-^5^I_8_). Electrons occupying the ^5^F_5_ energy level can also transition downward through the ^5^F_5_-^5^I_6_ (1454 nm) and ^5^F_5_-^5^I_7_ (755 nm) ways. Electron relaxation from ^5^I_4_ to the ^5^I_5_ energy level, followed by a transition to ^5^I_7_, releases near-infrared fluorescence at 1630 nm. The existence of cross-relaxation processes CR1, CR2, and CR3 induces quenching in the concentrated doped Ho-UCNPs, resulting in attenuated luminescence across all bands. The UC emission under laser irradiation at 2000 nm exhibits similarities. However, the UC process becomes more intricate, progressing to a four-photon or even five-photon process, leading to increased excitation energy loss. Consequently, the emission at 425 nm is not observed. Additionally, a notable emission at 1200 nm occurs in the near-infrared region from Ho-UCNPs when excited at 2000 nm. Er-UCNPs underwent irradiation with a 1520 nm laser, preferentially populating the ^4^I_13/2_ energy level. Subsequently, the red (660 nm: ^4^F_9/2_-^4^I_15/2_), green (550 nm: ^2^H_11/2_/^4^S_3/2_-^4^I_15/2_), blue (485 nm: ^4^F_3/2_/^4^F_5/2_-^4^I_15/2_), and purple (410 nm: ^2^H_9/2_-^4^I_15/2_) emission energy levels were gradually populated through three- and four-photon processes. The two-photon process was employed to populate the ^4^I_11/2_ and ^4^I_9/2_ energy levels. Cross-relaxation processes CR5 and CR6 induce a concentration quenching of visible luminescence, whereas CR4 and CR6 facilitate the population of the ^4^I_11/2_ energy level, resulting in strong 980 nm emission. Tm-UCNPs were stimulated by a 1215 nm laser, prompting the ground state electrons to transition to the ^3^F_3_ energy level through a two-photon UC process. This process resulted in luminescence at 700 nm (^3^F_3_-^3^H_6_), 808 nm (^3^H_4_-^3^H_6_), 1460 nm (^3^H_4_-^3^F_4_), and 1800 nm (^3^F_4_-^3^H_6_). Two cross-relaxation processes, CR7 and CR8, reduced the emission at 700 and 808 nm while intensifying the emission at 1800 nm in the concentrated doped Tm-UCNPs.


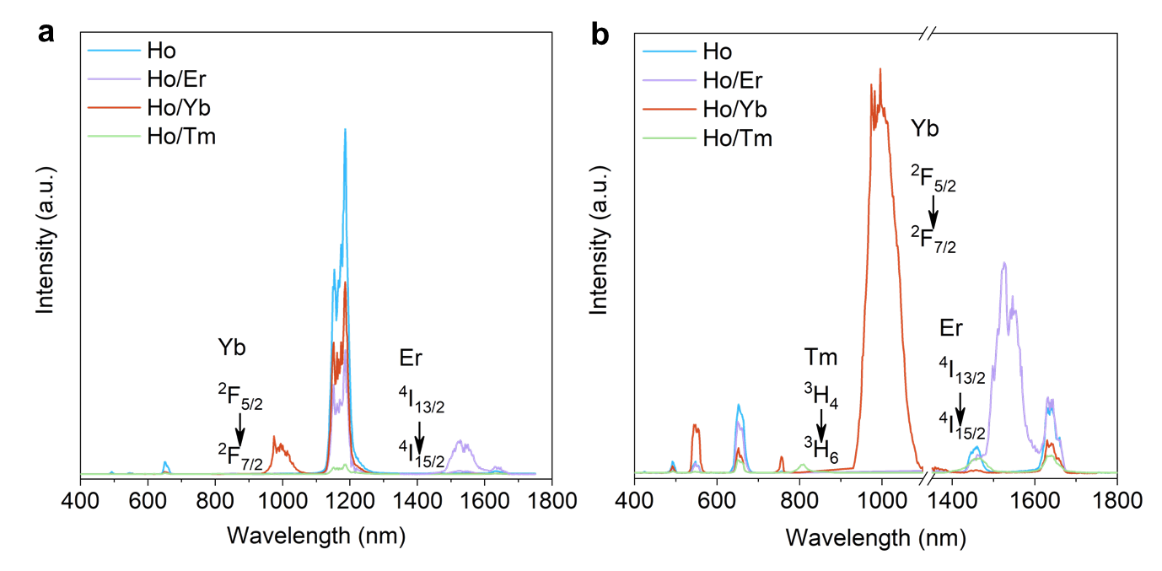


**Figure S5.** (a) Emission spectra of Ho-UCNPs, Ho/Er-UCNPs, Ho/Yb-UCNPs, Ho/Tm-UCNPs with 2000 nm excitation. (b) Emission spectra of Ho-UCNPs, Ho/Er-UCNPs, Ho/Yb-UCNPs, Ho/Tm-UCNPs with 1155 nm excitation.


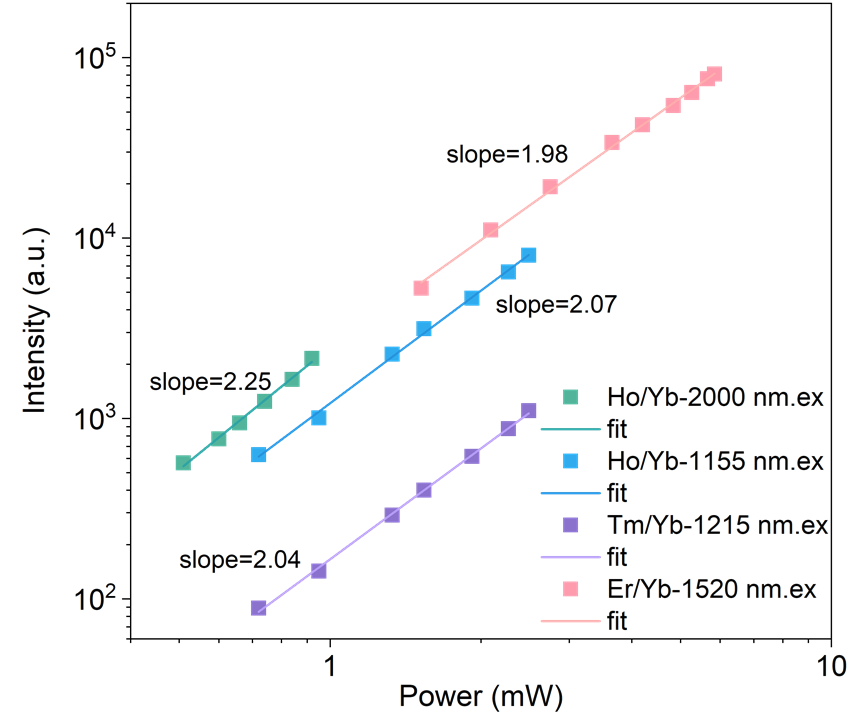


**Figure S6.** Variation of the emission intensity of Yb^3+^ ions at 980 nm with excitation optical power for Ln/Yb-UCNPs under 1155 nm, 1215 nm, 1520 nm, and 2000 nm excitation. The slopes derived from the linear fits are all approximately equal to 2, so the UC emission of Yb^3+^ ions can be considered a two-photon process.


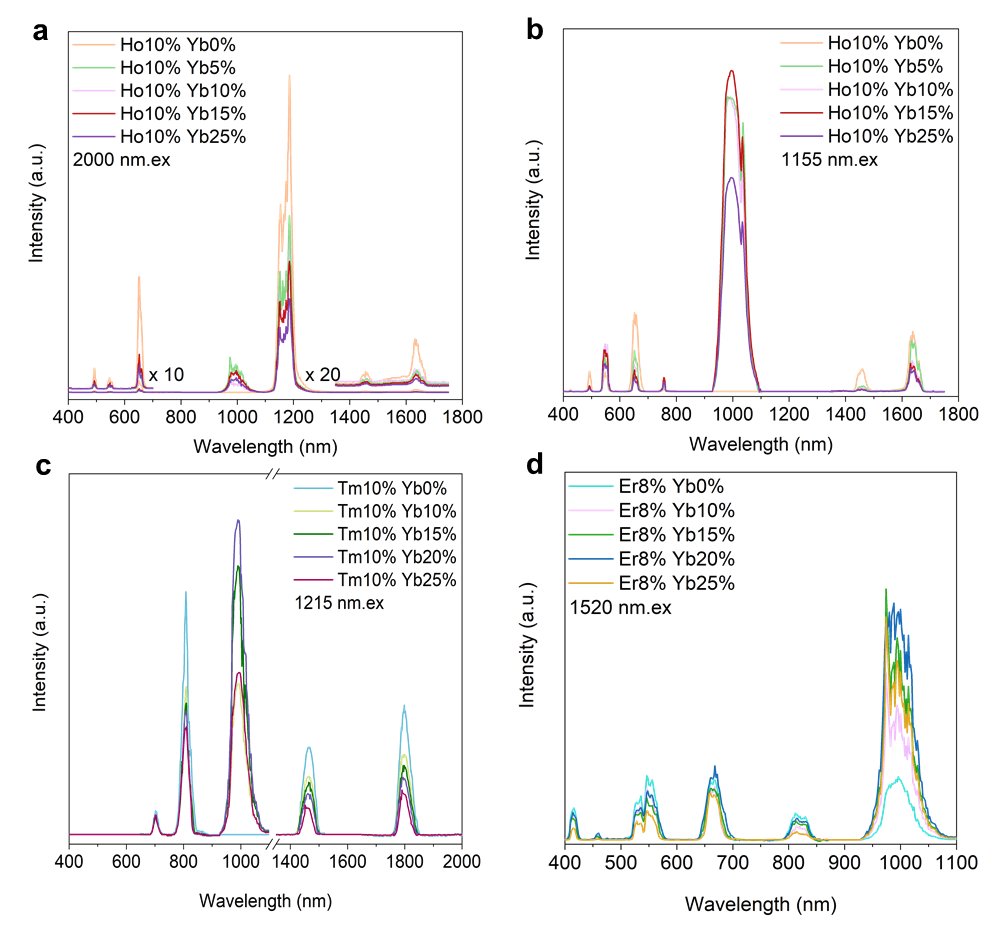


**Figure S7.** (a) Optimization of the emission spectra of Yb^3+^ doping concentration in NaYF_4_: Ho/Yb@ NaYF_4_ with an excitation wavelength of 2000 nm. (b) Optimization of the emission spectra of Yb^3+^ doping concentration in NaYF_4_: Ho/Yb@ NaYF_4_ with an excitation wavelength of 1155 nm. (c) Optimization of the emission spectra of Yb^3+^ doping concentration in NaYF_4_: Tm/Yb@ NaYF_4_ with an excitation wavelength of 1215 nm. (d) Optimization of the emission spectra of Yb^3+^ doping concentration in NaYF_4_: Er/Yb@ NaYF_4_ with an excitation wavelength of 1520 nm.


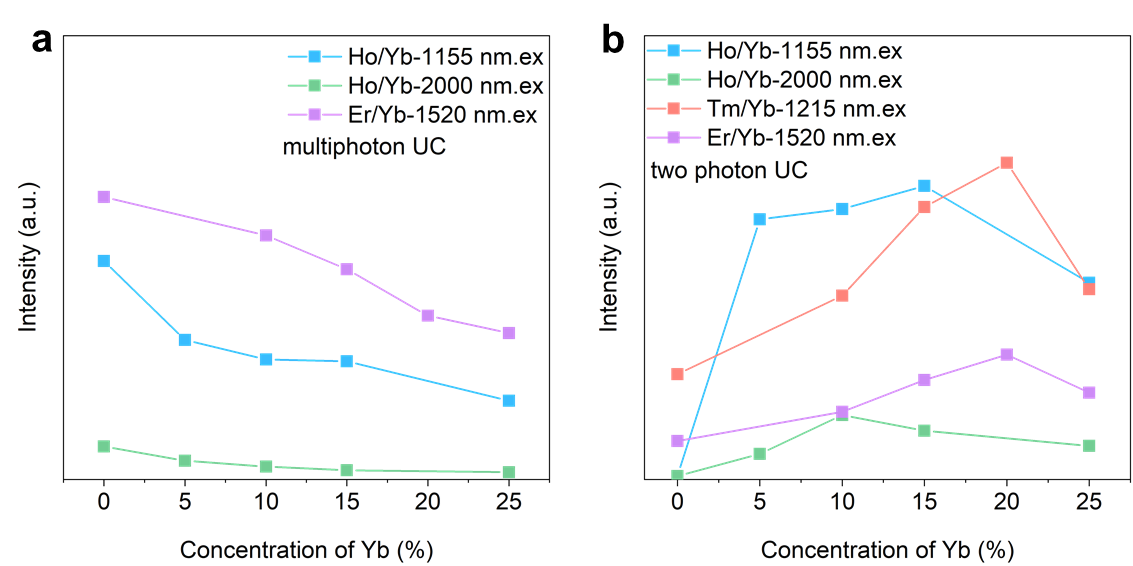


**Figure S8.** (a) Variation of the multiphoton UC intensity of Ln-UCNPs (Ln=Ho^3+^, Er^3+^) as a function of the doping concentration of Yb^3+^ ions. (b) Variation of the two photon UC intensity of Ln-UCNPs (Ln=Ho^3+^, Er^3+^, Tm^3+^) as a function of the doping concentration of Yb^3+^ ions.


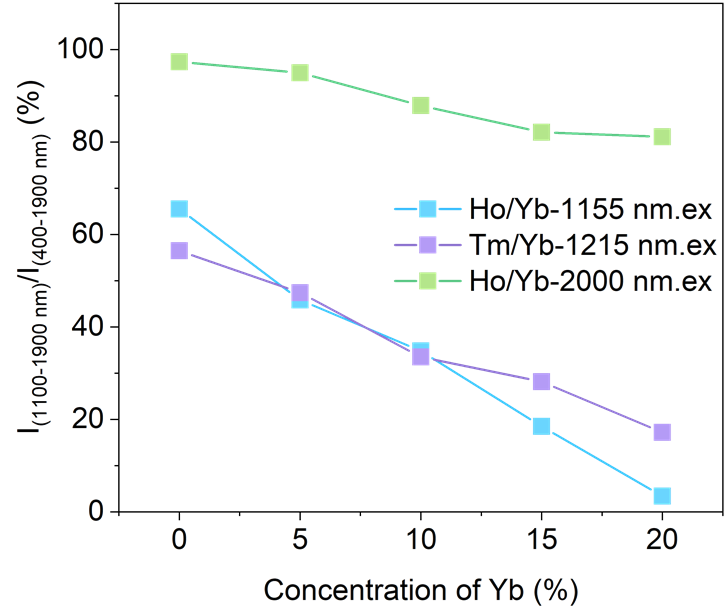


**Figure S9.** Integral intensity ratio I_(1100-1900 nm)_/I_(400-1900 nm)_ versus Yb ion doping concentration.


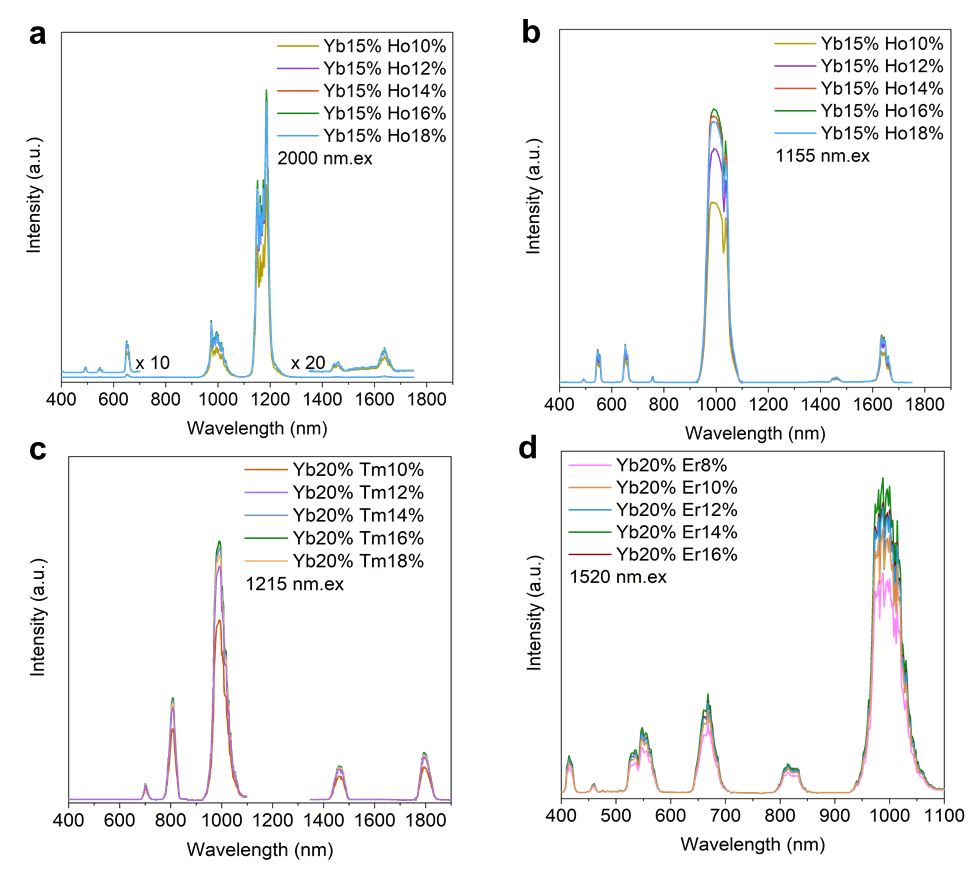


**Figure S10.** (a) The emission spectrum of Ho^3+^ doping continued in Ho/Yb-UCNPs, the excitation light is 2000 nm. (b) The emission spectrum of Ho^3+^ doping continued in Ho/Yb-UCNPs, the excitation light is 1155 nm. (c) The emission spectrum of Tm^3+^ doping continued in Tm/Yb-UCNPs, the excitation light is 1215 nm. (d) The emission spectrum of Er^3+^ doping continued in Er/Yb-UCNPs, the excitation light is 1520 nm.


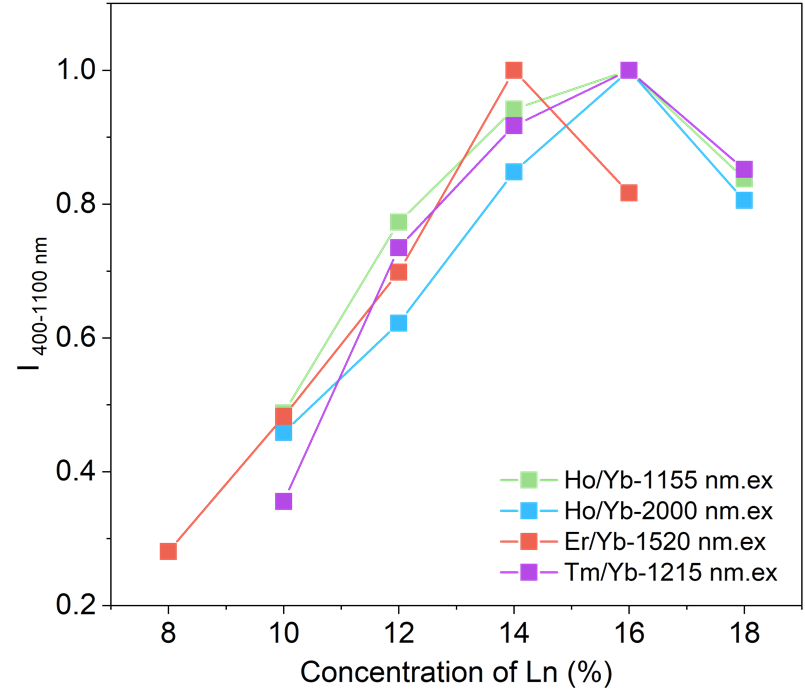


**Figure S11.** Trend of luminescence intensity with Ln^3+^ doping concentration from 400 to 1100 nm in Ln/Yb-UCNPs (Ln=Ho^3+^, Er^3+^, Tm^3+^).


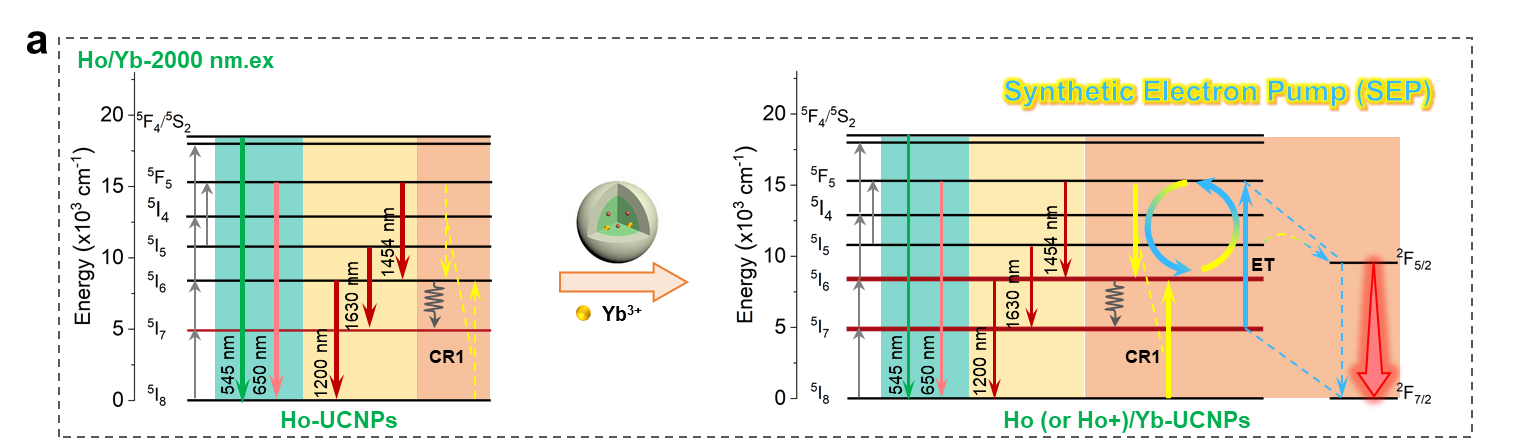


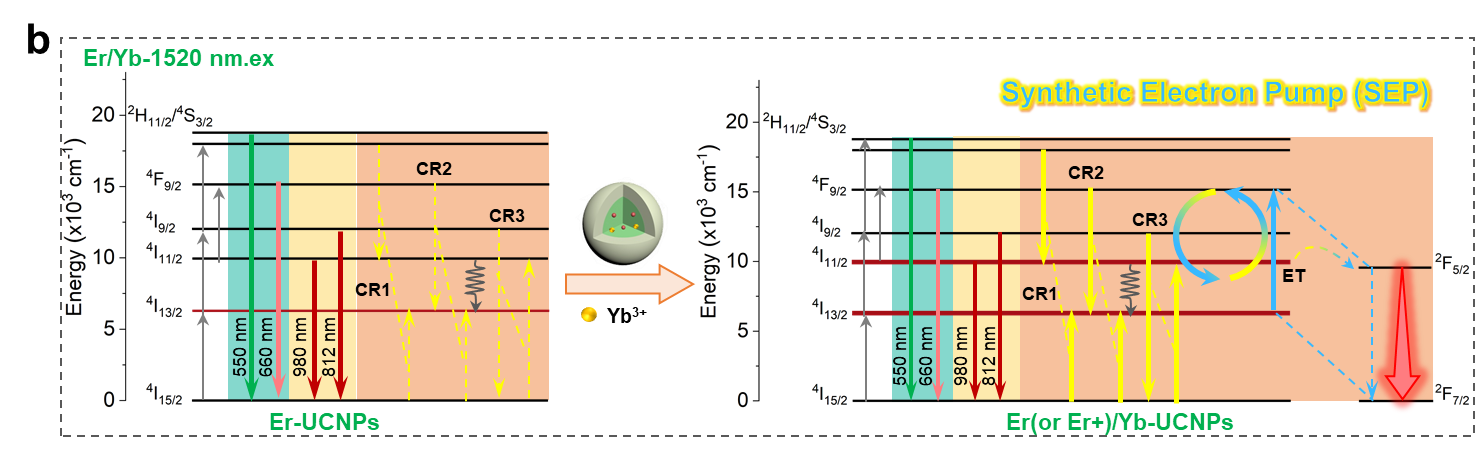


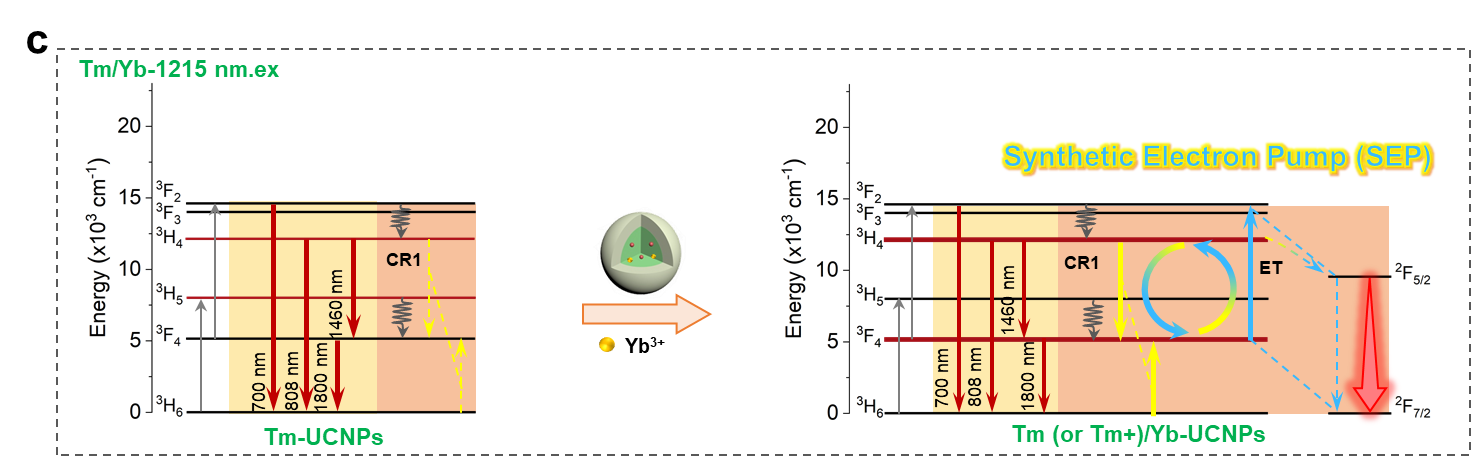
**Figure S12.** (a-c) A detailed description of the SEP effect of 2000 nm excitation of Ho/Yb-UCNPs, 1520 nm excitation of Er/Yb-UCNPs, and 1215 nm excitation of Tm/Yb-UCNPs. Ho^3+^, Er^3+^, and Tm^3+^ continually increase the electron population in the Yb^3+^ ^2^F_5/2_ energy level through the cross-relaxation process and the ET process, which promotes the enhancement of 980 nm luminescence. These steps are continuously repeated, ultimately forming a positive feedback loop. In this process, Yb^3+^ ions continuously receive energy from Ln^3+^, with a portion contributing to the positive feedback loop and another part used for their own luminescence.


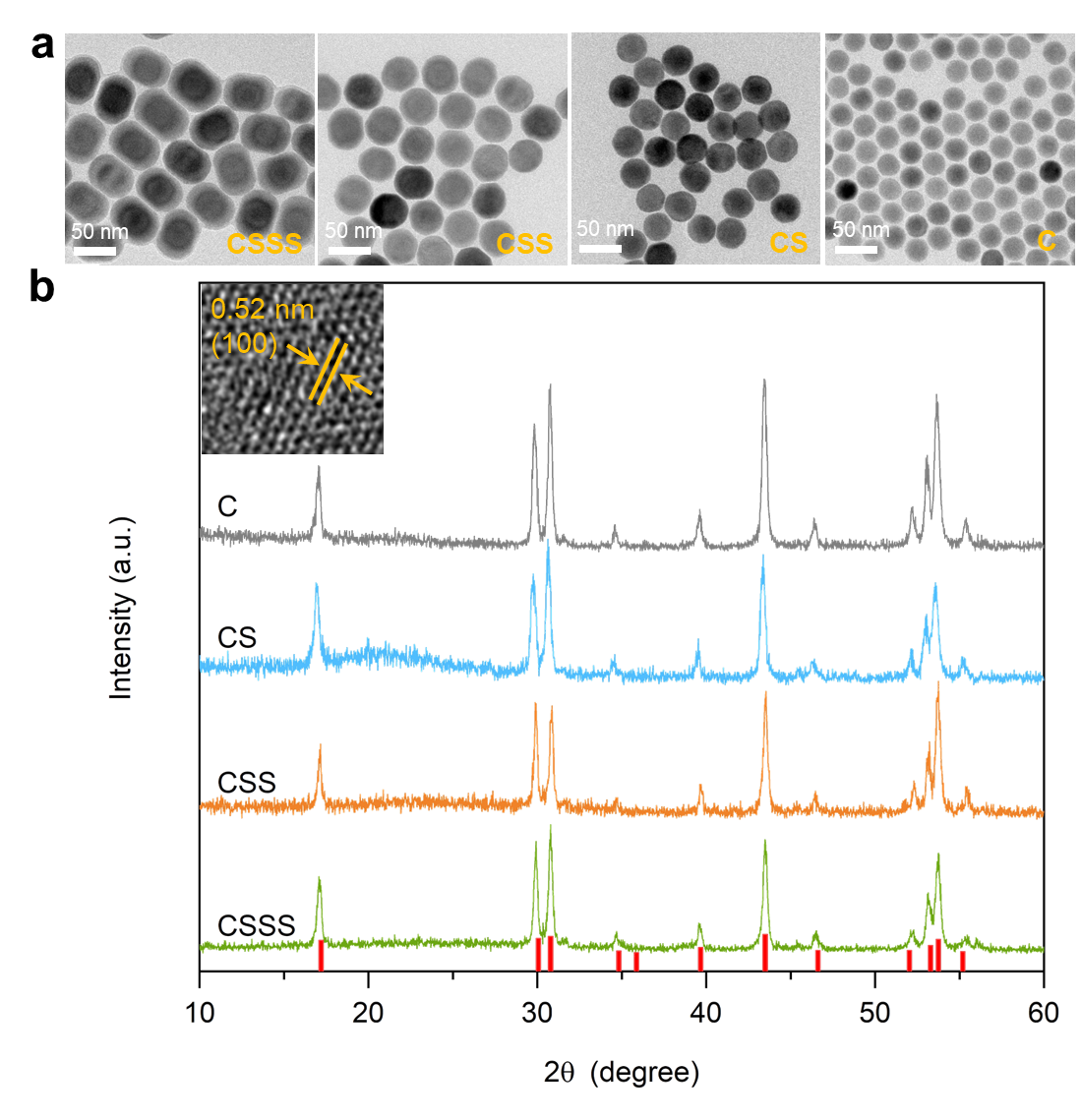


**Figure S13.** (a) Morphology characterization of as-synthesized C (core), CS (core-shell), CSS (core-shell-shell) and CSSS (core-shell-shell-shell) samples by transmission electron microscopy (TEM). (b) The XRD diffraction patterns of the synthesized C (core), CS (core-shell), CSS (core-shell-shell), and CSSS (core-shell-shell-shell), and the inset shows the high-resolution transmission electron microscope image of CSSS.


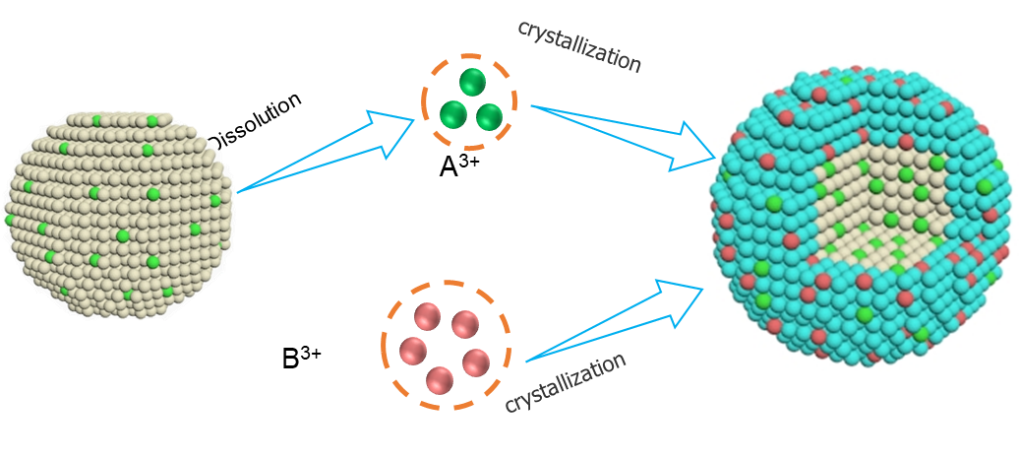


**Figure S14.** (a) Solvation recrystallization model for the synthesis of core-shell structured UCNPs.


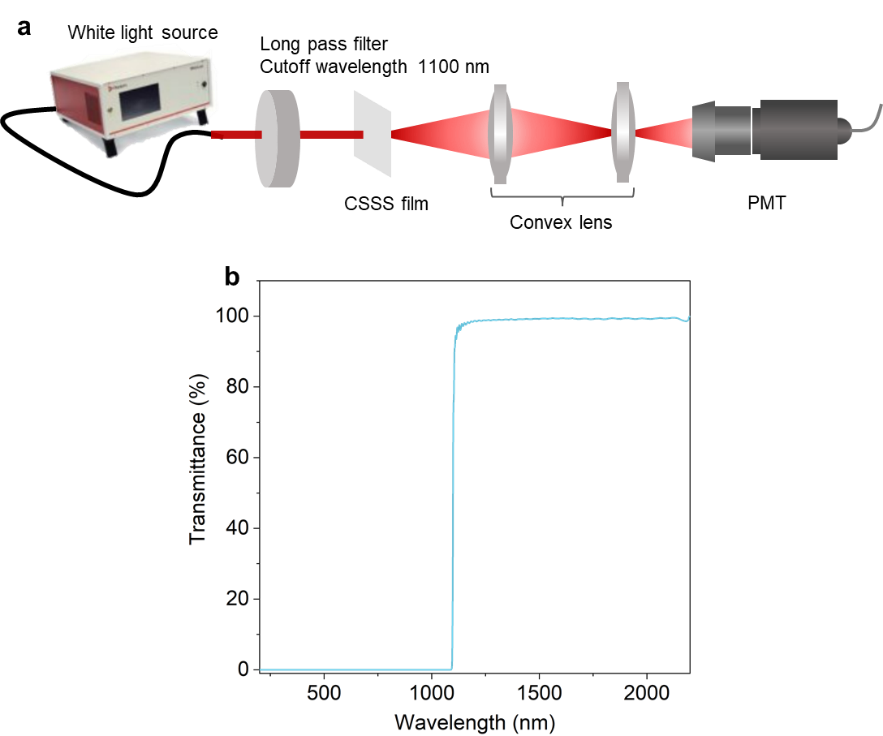


**Figure S15.** (a) Schematic experimental setup for optical characterization of upconversion properties of CSSS nanoparticles under broad-band near-infrared excitation in the range of 1100-2400 nm. (b) Measured transmittance spectrum of the long-pass filters (LP F2 (Supplier: Thorlabs; Model umber: FELH1100)) with cutoff wavelength of 1100 nm.


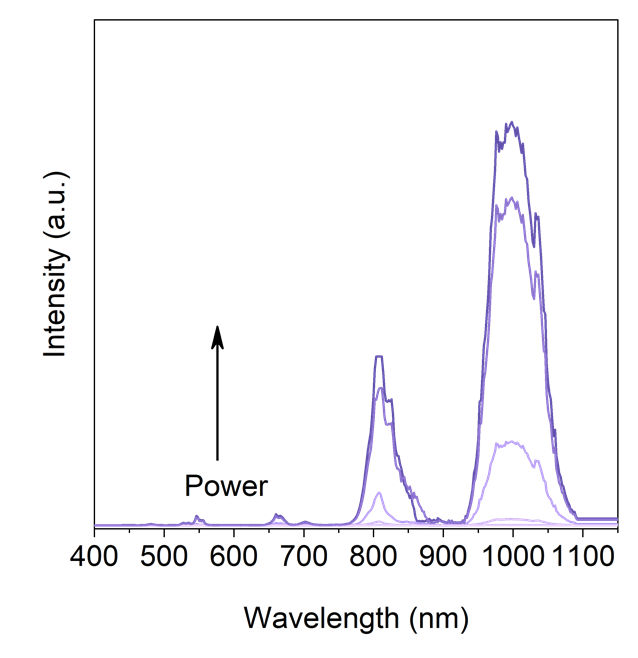


**Figure S16.** Emission spectra of the synthesized CSSS samples at different power density excitation conditions with excitation light of 1100–2400 nm were obtained.


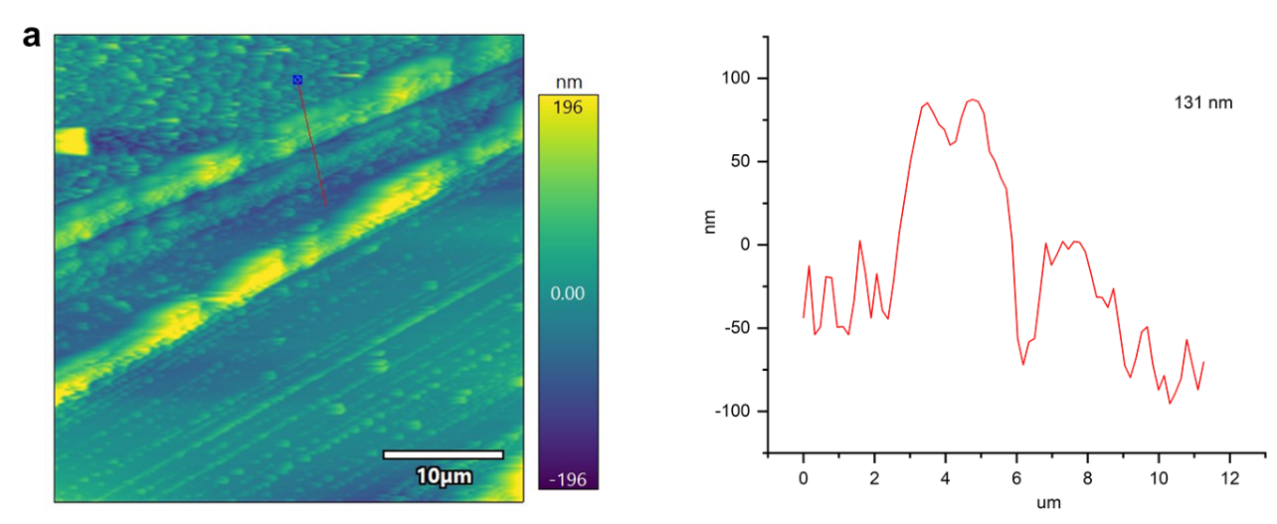


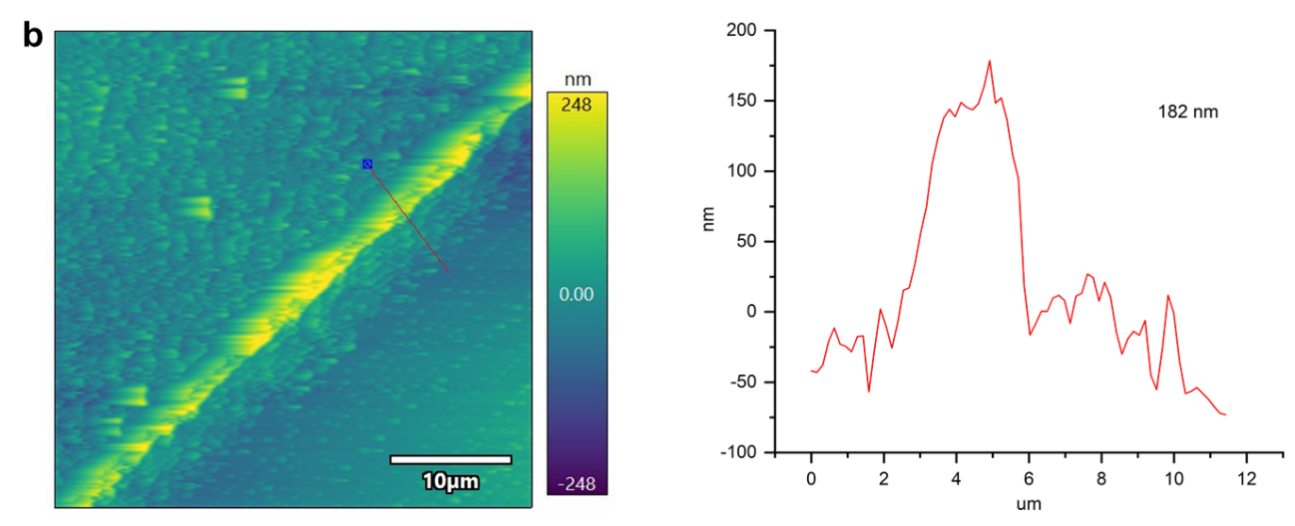


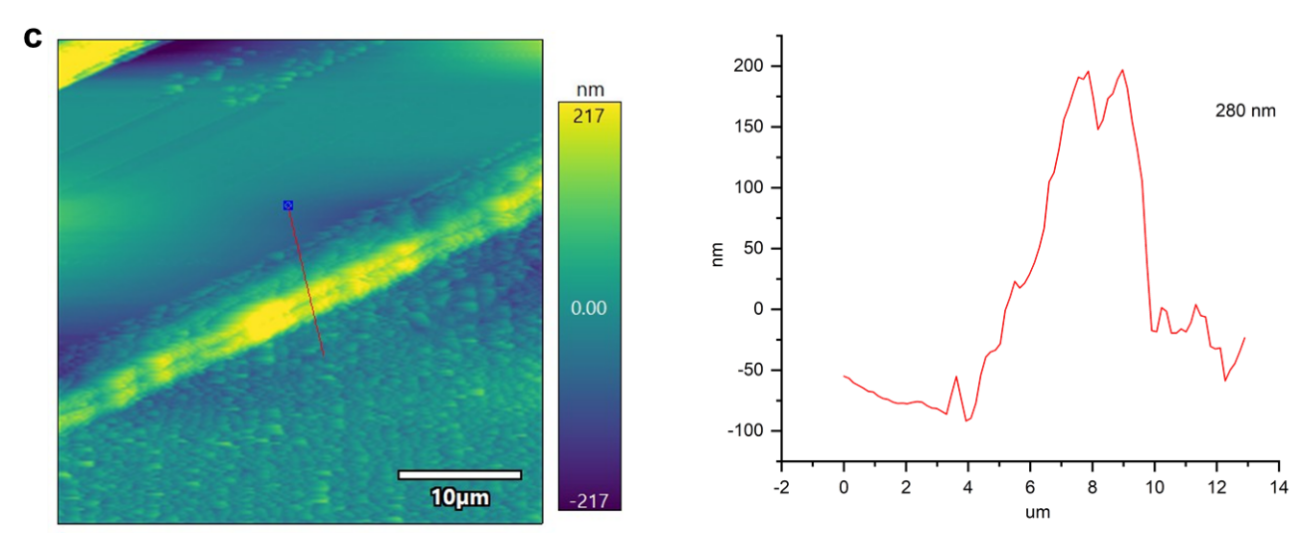


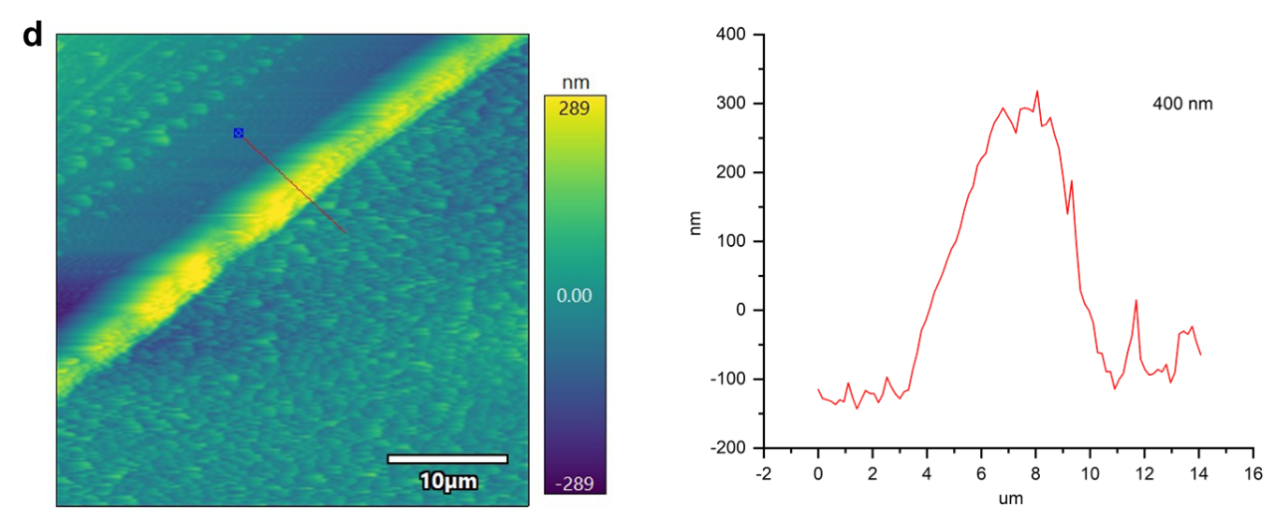


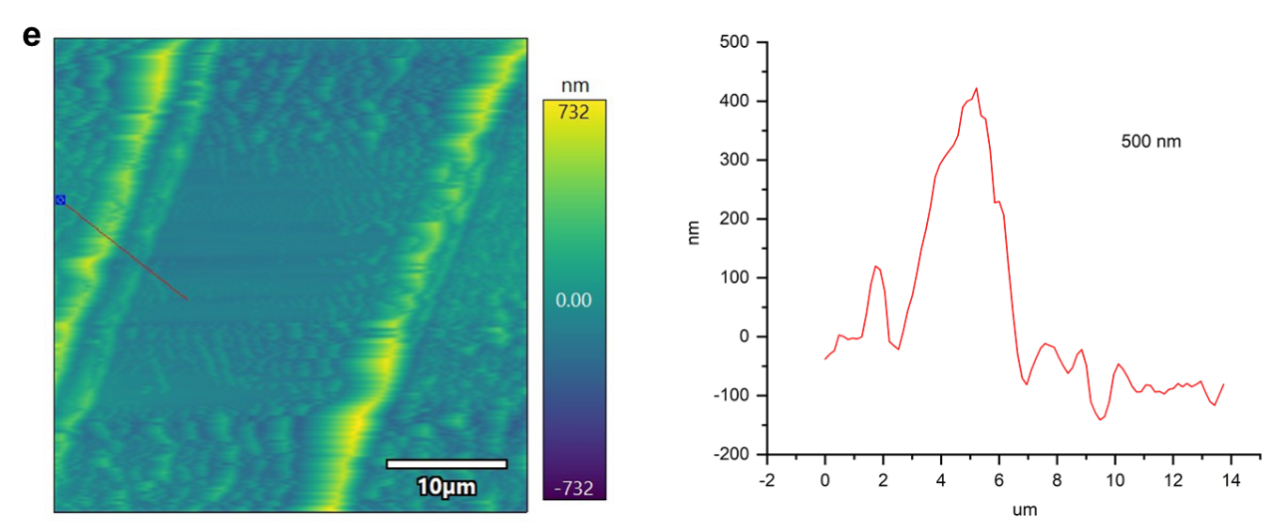


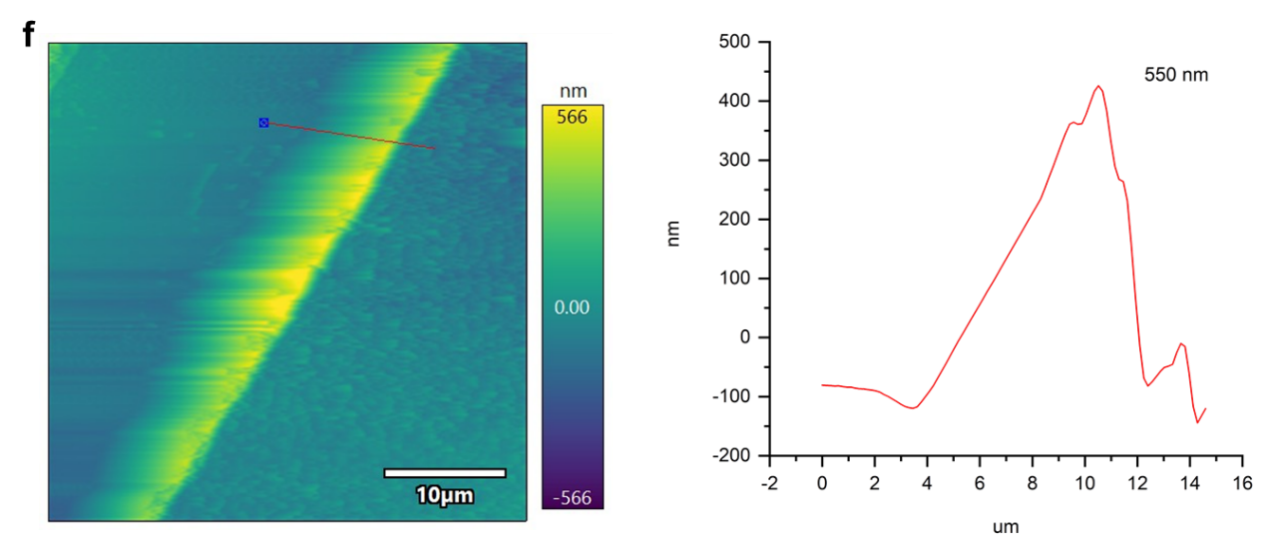


**Figure S17.** (a-f) AFM images thickness of CSSS film.


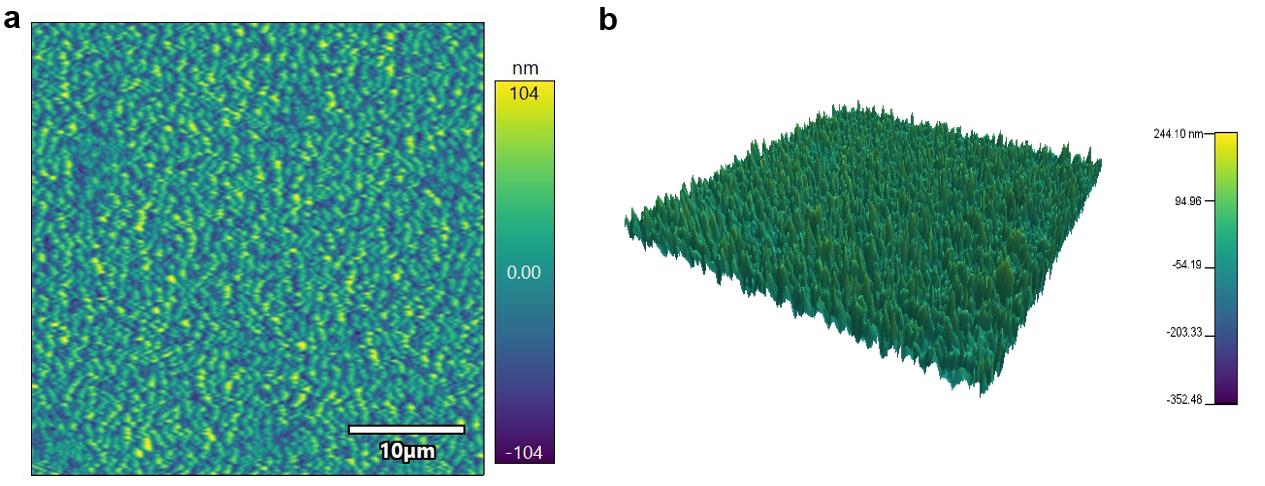
**Figure S18.** (a) Surface topography of CSSS film. (b) 3D AFM images of surface topography.


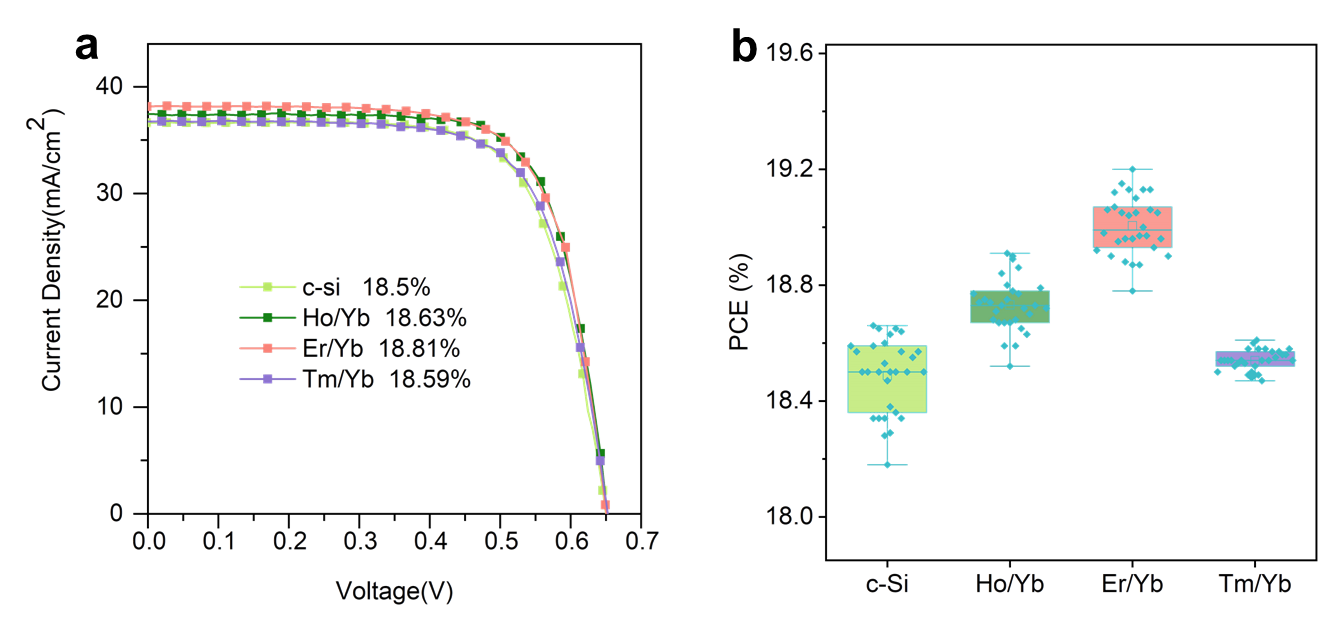


**Figure S19.** (a) I-V curves of c-Si solar cells coated with films doped with NaYF_4_: Ln/Yb (Ln=Ho/Er/Tm). (b) PCE distributions of c-Si solar cell coated with films doped with NaYF_4_: Ln/Yb (Ln=Ho/Er/Tm).


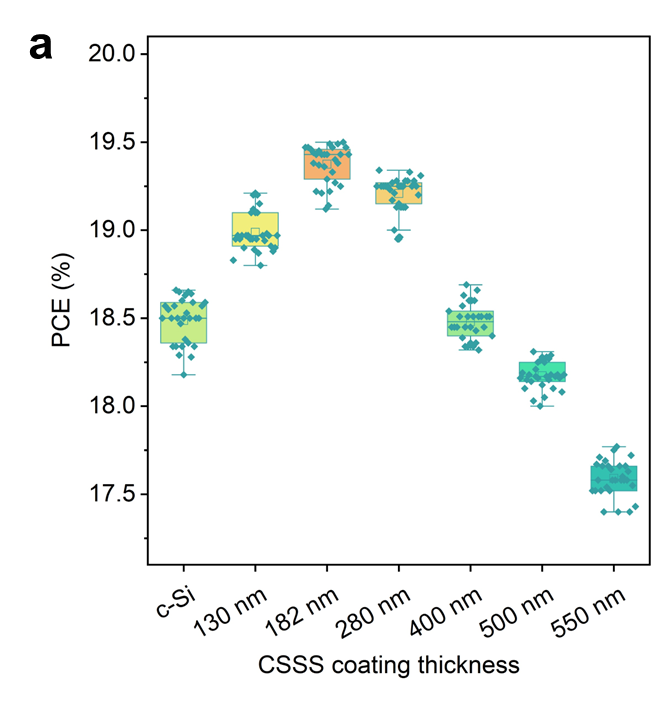


**Figure S20.** PCE distributions of c-Si solar cell coated with different thickness of CSSS samples. The number of each kind of CSSS coated solar cell was ten. The each of the CSSS coated solar cell was measured at three different areas and the distribution can be calculated based on the 30 PCE data.


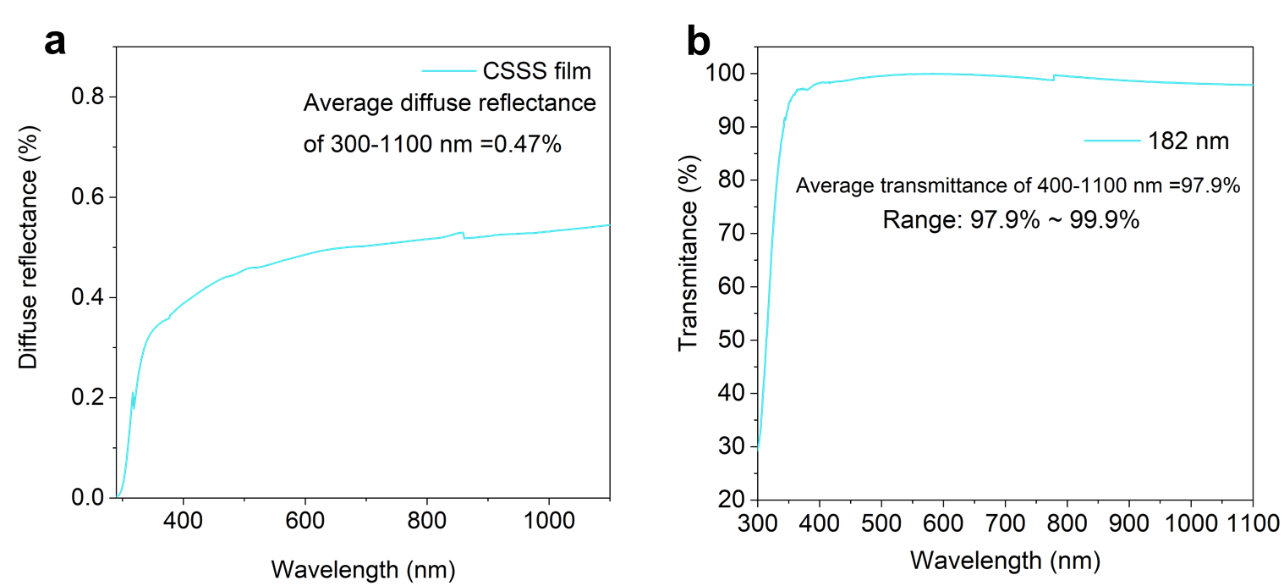
 **Figure S21.** (a) Diffuse reflection spectrum of the CSSS film with a thickness of 182 nm. (b) Transmission spectrum of the 182 nm- thick CSSS film. The results show that the film exhibit high quality, with minimal loss of incident light.
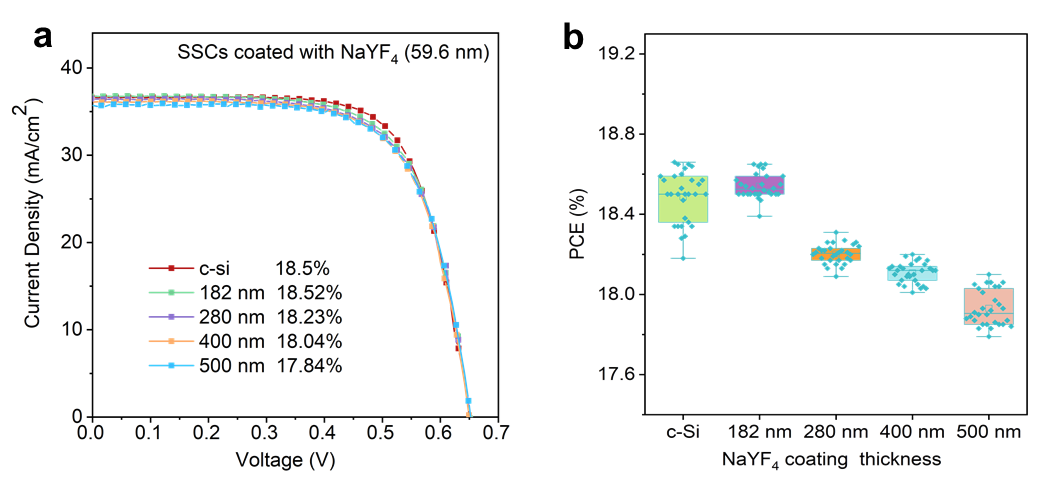


**Figure S22.** (a) I-V curves of c-Si solar cells coated with undoped NaYF_4_ nanoparticles (59.6 nm in diameter). (b) PCE distributions of c-Si solar cells coated with of NaYF_4_ (59.6 nm in diameter) films of different thickness.

**
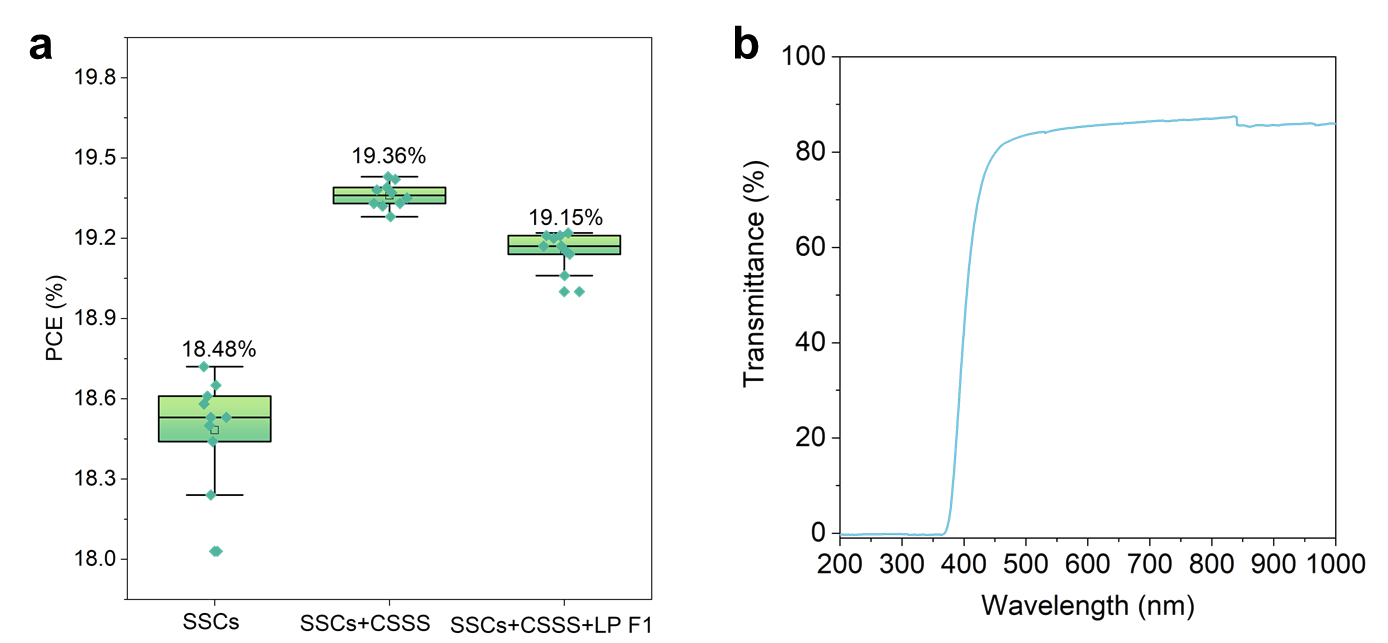
**

**Figure S23.** (a) The PCE distributions of SSC without CSSS film and SSC with CSSS film were statistically tested. The PCE distribution of the SSC coated with CSSS film when filtered by a low-pass filter with a cutoff wavelength of 400 nm was also statistically tested. The test irradiation conditions were AM 1.5G standard sunlight. (b) The transmittance of two low-pass filters (LP F1) with cutoff wavelengths of 400 nm was measured.

**
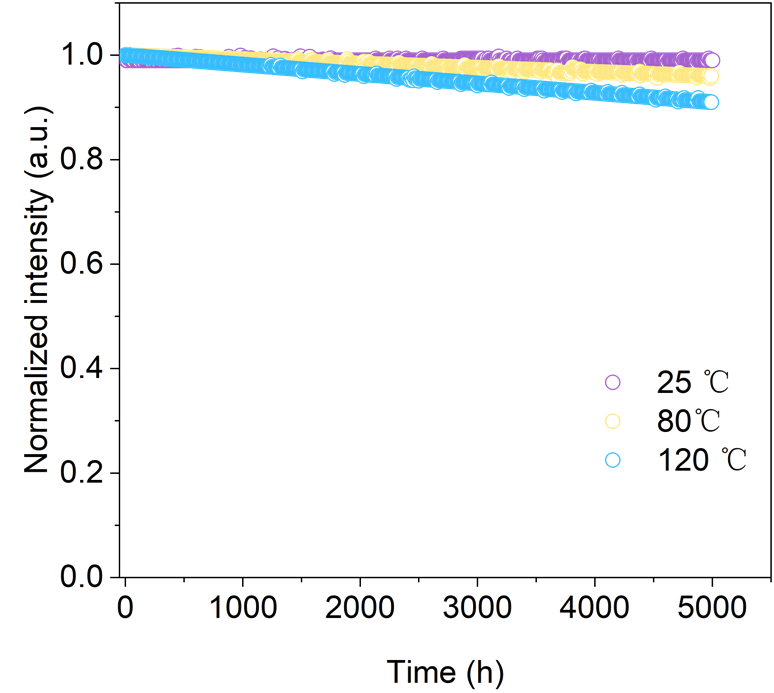
**

**Figure S24.** Temperature stability curves of CSSS coated SSCs.

**
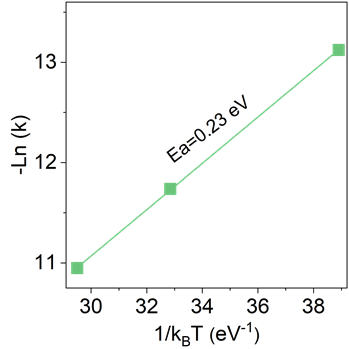
**

**Figure S25.** Natural logarithm of the degradation rate k versus 1/k_B_T, where T is the aging temperature.

**Supplementary Table S2:** Summarizes the excitation and emission wavelengths of up-conversion materials used in photovoltaic applications.

| **Dopant ions** | **Host materials** | **Excitation (nm)** | **Emission ( nm)** | **Ref.** |
| --- | --- | --- | --- | --- |
| Er^3+^ | Y_2_O_3_ | 980 | 525, 550, 660 | ^1^ |
| Er^3+^ | NaYF_4_ | 1523 | 550, 660, 800, 980 | ^2^ |
| Er^3+^ | NaYF_4_ | 1523 | 545, 670, 800, 980 | ^3^ |
| Er^3+^ | CaF_2_ | 1550 | 660, 980 | ^4^ |
| Er^3+^ | Y_2_O_3_ | 1538 | 562, 659, 801, 987 | ^5^ |
| Er^3+^-Yb^3+^ | Y_6_W_2_O_15_ | 973 | 516-570 | ^6^ |
| Er^3+^-Yb^3+^ | NaYF_4_ | 980 | 525, 540, 655 | ^7,8^ |
| Er^3+^-Yb^3+^ | NaYF_4_ | 980 | 522, 540, 653 | ^9^ |
| Er^3+^-Yb^3+^ | NaYF_4_ | 980 | 524, 540, 660 | ^10^ |
| Er^3+^-Yb^3+^ | Glass ceramic containing NaYF_4_ nanocrystals | 980 | 520, 538, 656 | ^11^ |
| Er^3+^-Yb^3+^ | LaF_3_ | 980 | 543, 655 | ^12^ |
| Er^3+^-Yb^3+^ | NaYF_4_ | 980 | 510-570,640-680 | ^13^ |
| Er^3+^-Yb^3+^ | YF_3_ | 975 | 510 - 560 | ^14^ |
| Tm^3+^-Yb^3+^ | Lu_2_O_3_ | 980 | 476, 653 | ^15^ |
| Ho^3+^-Yb^3+^ | Fluoroindate glass | 1155 | 550, 650, 750, 905, 980 | ^16^ |
| Ho^3+^-Yb^3+^ | Y_2_BaZnO_5_ | 986 | 545 | ^17^ |
| Ho^3+^ | NaYF_4_:Ho@ NaYF_4_ | 1155, 2000 | 490, 545, 650, 1454, 1630 | This work |
| Ho^3+^, Er^3+^, Tm^3+^, Yb^3+^ | CSSS | 1155, 1215, 1520, 1750, 2000 | 400-2100 | This work |

**Supplementary Table S3:** Summary of up-conversion materials in improving the performance of silicon solar cell type. The rightmost column corresponds to the vertical coordinate in Fig. 4f in the main text.

| **Sample** | **Solar cell** | **Intensity of irradiation** | **Enhancement Current density (mA cm^-2^)** | **Ref.** |  |
| --- | --- | --- | --- | --- | --- |
| β-NaYF_4_: 25% Er^3+^ | Bifacial silicon solar cell | 94-suns | 9.4 mA cm^-2^ | ^18^ |  |
| Bi_4_Ti_3_O_12_: Er^3+^ | silicon solar cell | AM 1.5G | --- | ^19^ | h |
| (Er, Ho)_2_O_3_ | silicon solar cell | 16-suns | --- | ^20^ | g |
| β-NaYF_4_: Er^3+^ | silicon solar cell | 78-suns | 2.2 mA cm^-2^ | ^21^ | d |
| Gd_2_O_2_S:10%Er^3+^ powders | Bifacial silicon solar cell | 94-suns | 8.2 mA cm^-2^ | ^18^ | b |
| BaY_2_F_8_:30%Er^3+^ monocrystalline | Bifacial silicon solar cell | 94±17 suns | 17.2±3.0 mA cm^-2^ | ^22^ | a |
| NaYF_4_: 20% Er^3+^ | Bifacial silicon solar cell | 1523 nm Laser | EQE=3.4% | ^23^ |  |
| NaYF_4_:Er^3+^ phosphors | Bifacial silicon solar cell | 1523 nm Laser (5.1 mW) | EQE=2.5±0.2%  IQE=3.8% | ^2^ |  |
| NaYF_4_: 20% Er^3+^ | Bifacial silicon solar cell | 1522 nm (1090 W cm^-2^) | EQE=0.34% | ^3^ |  |
| β-NaYF4: 25% Er^3+^ | Bifacial silicon solar cell | 77/19/82-suns | 4.03 mA cm^-2^ | ^24^ | c/e/f |
| β-NaYF4: 25% Er^3+^ powders | Bifacial silicon solar cell | 50-suns | 3.7 mA cm^-2^ | ^18^ |  |
| CSSS- films | Single sided silicon solar cell | AM 1.5G | 2 mA cm^-2^ | This work |  |

**Reference**

1 Wang, J. *et al.* Application of Y_2_O_3_: Er^3+^ nanorods in dye-sensitized solar cells. *ChemSusChem* **5**, 1307-1312 (2012). https://doi.org/10.1002/cssc.201100596

2 Shalav, A., Richards, B. S., Trupke, T., Krämer, K. W. & Güdel, H. U. Application of NaYF_4_:Er^3+^ up-converting phosphors for enhanced near-infrared silicon solar cell response. *Appl. Phys. Lett.* **86** (2005). https://doi.org/10.1063/1.1844592

3 Fischer, S. *et al.* Enhancement of silicon solar cell efficiency by upconversion: Optical and electrical characterization. *Journal of Applied Physics* **108** (2010). https://doi.org/10.1063/1.3478742

4 Ivanova, S. *et al.* Upconversion luminescence dynamics of Er-doped fluoride crystals for optical converters. *Journal of Luminescence* **128**, 914-917 (2008). https://doi.org/10.1016/j.jlumin.2007.11.031

5 Wang, X., Yan, X. & Kan, C. Controlled synthesis and optical characterization of multifunctional ordered Y_2_O_3_: Er^3+^ porous pyramid arrays. *Journal of Materials Chemistry* **21** (2011). https://doi.org/10.1039/c0jm03761c

6 Lin, H.-Y. *et al.* Investigation of Green Up-Conversion Behavior in Y_6_W_2_O_15_:Yb^3+^, Er^3+^ Phosphor and its Verification in 973-nm Laser-Driven GaAs Solar Cell. *J. Am. Chem. Soc.* **95**, 3172-3179 (2012). https://doi.org/10.1111/j.1551-2916.2012.05281.x

7 Zhang, X. D. *et al.* Synthesis of NaYF_4_: Yb, Er nanocrystals and its application in silicon thin film solar cells. *physica status solidi c* **7**, 1128-1131 (2010). https://doi.org/10.1002/pssc.200982762

8 de Wild, J., Meijerink, A., Rath, J. K., van Sark, W. G. J. H. M. & Schropp, R. E. I. Towards upconversion for amorphous silicon solar cells. *Solar Energy Materials and Solar Cells* **94**, 1919-1922 (2010). https://doi.org/10.1016/j.solmat.2010.06.006

9 de Wild, J., Rath, J. K., Meijerink, A., van Sark, W. G. J. H. M. & Schropp, R. E. I. Enhanced near-infrared response of a-Si:H solar cells with β-NaYF_4_:Yb^3+^ (18%), Er^3+^ (2%) upconversion phosphors. *Solar Energy Materials and Solar Cells* **94**, 2395-2398 (2010). https://doi.org/10.1016/j.solmat.2010.08.024

10 Li, Z. Q. *et al.* Core/shell structured NaYF_4_:Yb^3+^/Er^3+^/Gd^+3^ nanorods with Au nanoparticles or shells for flexible amorphous silicon solar cells. *Nanotechnology* **23**, 025402 (2012). https://doi.org/10.1088/0957-4484/23/2/025402

11 Zhao, S. *et al.* Er^3+^/Yb^3+^ codoped oxyfluoride borosilicate glass ceramic containing NaYF4 nanocrystals for amorphous silicon solar cells. *Materials Letters* **65**, 2407-2409 (2011). https://doi.org/10.1016/j.matlet.2011.04.084

12 Shan, G. B. & Demopoulos, G. P. Near-infrared sunlight harvesting in dye-sensitized solar cells via the insertion of an upconverter-TiO_2_ nanocomposite layer. *Adv Mater* **22**, 4373-4377 (2010). https://doi.org/10.1002/adma.201001816

13 Shan, G. B., Assaaoudi, H. & Demopoulos, G. P. Enhanced performance of dye-sensitized solar cells by utilization of an external, bifunctional layer consisting of uniform beta-NaYF_4_: Er^3+^/Yb^3+^ nanoplatelets. *ACS Appl. Mater. Interfaces* **3**, 3239-3243 (2011). https://doi.org/10.1021/am200537e

14 Wang, H. Q., Batentschuk, M., Osvet, A., Pinna, L. & Brabec, C. J. Rare-earth ion doped up-conversion materials for photovoltaic applications. *Adv Mater* **23**, 2675-2680 (2011). https://doi.org/10.1002/adma.201100511

15 Li, Q. *et al.* Enhancing photovoltaic performance of dye-sensitized solar cell by rare-earth doped oxide of Lu_2_O_3_: Tm^3+^, Yb^3+^. *Electrochim. Acta.* **56**, 4980-4984 (2011). https://doi.org/10.1016/j.electacta.2011.03.125

16 Lahoz, F. *et al.* Upconversion mechanisms in rare-earth doped glasses to improve the efficiency of silicon solar cells. *Solar Energy Materials and Solar Cells* **95**, 1671-1677 (2011). https://doi.org/10.1016/j.solmat.2011.01.027

17 Adikaari, A. A. D. *et al.* Near infrared up-conversion in organic photovoltaic devices using an efficient Yb^3+^, Ho^3+^ Co-doped Ln_2_BaZnO_5_ (Ln = Y, Gd) phosphor. *Journal of Applied Physics* **111** (2012). https://doi.org/10.1063/1.4704687

18 Fischer, S. *et al.* Upconversion solar cell measurements under real sunlight. *Optical Materials* **84**, 389-395 (2018). https://doi.org/10.1016/j.optmat.2018.05.072

19 Fuentes, S., Vega, M., Aguayo, M. & Morales, P. Upconversion of Bi_4_Ti_3_O_12_: Er and its evaluation in silicon solar cell yield. *Materials Letters* **296**, 129889-129889 (2021). https://doi.org/10.1016/j.matlet.2021.129889

20 Ghazy, A. *et al.* Luminescent (Er, Ho)_2_O_3_ thin films by ALD to enhance the performance of silicon solar cells. *Solar Energy Materials and Solar Cells* **219**, 110787-110787 (2021). https://doi.org/10.1016/j.solmat.2020.110787

21 Fischer, S. *et al.* Upconverter Silicon Solar Cell Devices for Efficient Utilization of Sub-Band-Gap Photons Under Concentrated Solar Radiation. *IEEE J PHOTOVOLT* **4**, 183-189 (2014). https://doi.org/10.1109/jphotov.2013.2282744

22 Fischer, S., Favilla, E., Tonelli, M. & Goldschmidt, J. C. Record efficient upconverter solar cell devices with optimized bifacial silicon solar cells and monocrystalline BaY_2_F_8_:30% Er^3+^ upconverter. *Solar Energy Materials and Solar Cells* **136**, 127-134 (2015). https://doi.org/10.1016/j.solmat.2014.12.023

23 Richards, B. S. & Shalav, A. Enhancing the Near-Infrared Spectral Response of Silicon Optoelectronic Devices via Up-Conversion. *IEEE Transactions on Electron Devices* **54**, 2679-2684 (2007). https://doi.org/10.1109/ted.2007.903197

24 Fischer, S., Fröhlich, B., Steinkemper, H., Krämer, K. W. & Goldschmidt, J. C. Absolute upconversion quantum yield of β-NaYF_4_ doped with Er^3+^ and external quantum efficiency of upconverter solar cell devices under broad-band excitation considering spectral mismatch corrections. *Solar Energy Materials and Solar Cells* **122**, 197-207 (2014). https://doi.org/10.1016/j.solmat.2013.12.001
